# Differences between joint-space and musculoskeletal estimations of metabolic rate time profiles

**DOI:** 10.1371/journal.pcbi.1008280

**Published:** 2020-10-28

**Authors:** Arash Mohammadzadeh Gonabadi, Prokopios Antonellis, Philippe Malcolm

**Affiliations:** 1 Department of Biomechanics and Center for Research in Human Movement Variability, University of Nebraska at Omaha, Omaha, Nebraska, United States of America; 2 Rehabilitation Engineering Center, Institute for Rehabilitation Science and Engineering, Madonna Rehabilitation Hospitals, Lincoln, Nebraska, United States of America; Ecole Polytechnique Fédérale de Lausanne, SWITZERLAND

## Abstract

Motion capture laboratories can measure multiple variables at high frame rates, but we can only measure the average metabolic rate of a stride using respiratory measurements. Biomechanical simulations with equations for calculating metabolic rate can estimate the time profile of metabolic rate within the stride cycle. A variety of methods and metabolic equations have been proposed, including metabolic time profile estimations based on joint parameters. It is unclear whether differences in estimations are due to differences in experimental data or due to methodological differences. This study aimed to compare two methods for estimating the time profile of metabolic rate, within a single dataset. Knowledge about the consistency of different methods could be useful for applications such as detecting which part of the gait cycle causes increased metabolic cost in patients. Here we compare estimations of metabolic rate time profiles using a musculoskeletal and a joint-space method. The musculoskeletal method was driven by kinematics and electromyography data and used muscle metabolic rate equations, whereas the joint-space method used metabolic rate equations based on joint parameters. Both estimations of changes in stride average metabolic rate correlated significantly with large changes in indirect calorimetry from walking on different grades showing that both methods accurately track changes. However, estimations of changes in stride average metabolic rate did not correlate significantly with more subtle changes in indirect calorimetry due to walking with different shoe inclinations, and both the musculoskeletal and joint-space time profile estimations did not correlate significantly with each other except in the most downward shoe inclination. Estimations of the relative cost of stance and swing matched well with previous simulations with similar methods and estimations from experimental perturbations. Rich experimental datasets could further advance time profile estimations. This knowledge could be useful to develop therapies and assistive devices that target the least metabolically economic part of the gait cycle.

## Author summary

Respiratory oxygen consumption measurements allow recording of the average metabolic cost of walking, but the slow rate of these measurements prevents assessing which part of the gait cycle has the highest metabolic cost. Simulation methods allow estimation of the time profile of metabolic cost within the gait cycle. We compared estimations of the metabolic cost of walking using a method that is based on kinematic and electromyography recordings from participants in conjunction with muscle metabolic rate equations and a method based on joint kinematics and kinetics. While both methods are able to accurately estimate large changes in the average metabolic cost from walking on different inclinations, the time profiles estimated by the two methods are different, indicating that estimations of the time profile of metabolic cost are dependent on the estimation method. Both estimation methods matched well with the results from experimental perturbation studies that suggest that the metabolic cost of the swing phase is approximately 10% to 20% of the metabolic cost of the entire gait cycle. Advances in estimations of the time profile of metabolic cost could lead to practical applications such as designing rehabilitation robots that assist specifically during the phase with the highest metabolic cost.

## Introduction

Would we be able to detect and understand the effects of gait impairments if we could capture only average metrics instead of time series? Motion capture laboratories can currently perform measurements of a person's movements (kinematics), forces (kinetics), and muscle electromyography (EMG) at hundreds of frames per second, which allows us to analyze the time profiles of these variables within the stride cycle. Energy from food is supplied to the human body in the form of chemical energy in the muscles (metabolic energy expenditure). This metabolic energy expenditure is one of the main determinants of the way we walk [1–5], and indirect calorimetry measurements are an important tool for understanding how increases in metabolic cost restrict the mobility of clinical populations [6–8]. In contrast to EMG and kinematic measurements, the metabolic cost can be measured only once per breath, and one needs to average several minutes of data to obtain a reliable average measurement [9]. As a result, we cannot directly measure which phases of the stride cycle have an increased metabolic cost in patient populations. Knowing such information would allow the design of assistive devices or therapies that specifically target phases of gait with increased metabolic cost.

The cost of walking can also be estimated using simulations with muscle metabolic rate equations [10–17]. These equations allow the time profile of the metabolic rate within the stride cycle to be computed. Umberger et al., [17] developed muscle metabolic rate equations that have been widely used [12,16,18–20], which divide muscle metabolic rate into components due to energy loss from heat production during activation and maintenance of activation of muscle fibers (activation-maintenance heat-rate), energy loss from heat production during shortening and lengthening of muscle fibers (shortening-lengthening heat-rate), and mechanical fiber work. A follow-up study from Umberger [20] applied muscle metabolic rate equations in a 2D model with 12 muscles per side that are each composed of a contractile, series elastic element, and parallel element (Hill-type muscles [21]). Umberger [20] used forward dynamics simulation to predict the effect of muscle excitations in a rigid body model. Initial conditions were based on motion capture data, and optimization was used to solve the redundancy problem where different muscle excitations can produce a possible gait pattern. The optimization identified the muscle excitation patterns that produced a cyclic gait, and that minimized a cost function based on the muscle metabolic rate equations [17] according to the assumption that humans move in a way that minimizes metabolic cost. Using the resulting muscle behaviors, Umberger [20] obtained the first detailed estimation of the time profile of metabolic rate during the walking stride cycle. Kim et al., [22] and Roberts et al., [23] argue that there are specific challenges associated with muscle models because they simulate only a subset of muscles and because complicated interactions between muscles and other tissues are difficult to simulate. They developed equations to estimate the time profile of metabolic rate as a function of joint parameters of a 3D full-body model [22,23]. Their equations also divide metabolic rate into subcomponents due to activation-maintenance, shortening-lengthening, and mechanical work. Finally, their model only uses joint moments and joint angular velocities as inputs in combination with coefficients that have been optimized based on experimental data from walking at different speeds.

Certain experimental studies have aimed to partition metabolic cost into functional roles, such as body weight support and center-of-mass acceleration [24,25], yet others have aimed to partition metabolic cost into different temporal phases including stance, step-to-step transition, and swing [26–29]. More specifically, the metabolic cost of the swing phase was estimated to range from 10% to 20% of the cost of the entire stride cycle based on experiments that involved assisting swing initiation with bungee cords [29] and increasing the body mass [27]. Another study measured that the metabolic cost of swinging one leg backward and forward while standing on the other leg is 35% of the total metabolic cost of walking [28]. By assuming that the cost of swinging the legs forward is one-half of the cost of swinging the legs in both directions, one could estimate that the cost of the swing phase is 17% of the metabolic rate of walking. It was estimated that the cost of the step-to-step transitions is 45% of the entire stride cycle by independently manipulating body mass and body weight with a weight vest and a body weight support harness [26]. Estimations of the temporal distribution of metabolic rate are not always consistent. Estimations of the relative cost of the swing phase derived from experimental studies (no more than 20% [27], 10% [29], ~17% [28]) are lower than estimations from simulation studies (29% [20] and 26% [19]). Estimations of the total cost of both double support phases (approximately 45%) from experiments with added mass and body weight support [26] are higher than those from certain simulation studies (27% [19,20]). The forward simulation from Umberger [20] predicts a higher cost for the weight-acceptance phase and a lower cost for the push-off phase compared to the joint-space estimation method from Roberts et al., [23]. These inconsistencies could be due to differences in the estimation methods [12,14,30] or differences in measurement data due to participant selection, walking conditions (e.g., speed, shoes), measurement errors (e.g., soft tissue artifacts, [31]), and marker placement artifacts [32].

**Fig 1:**
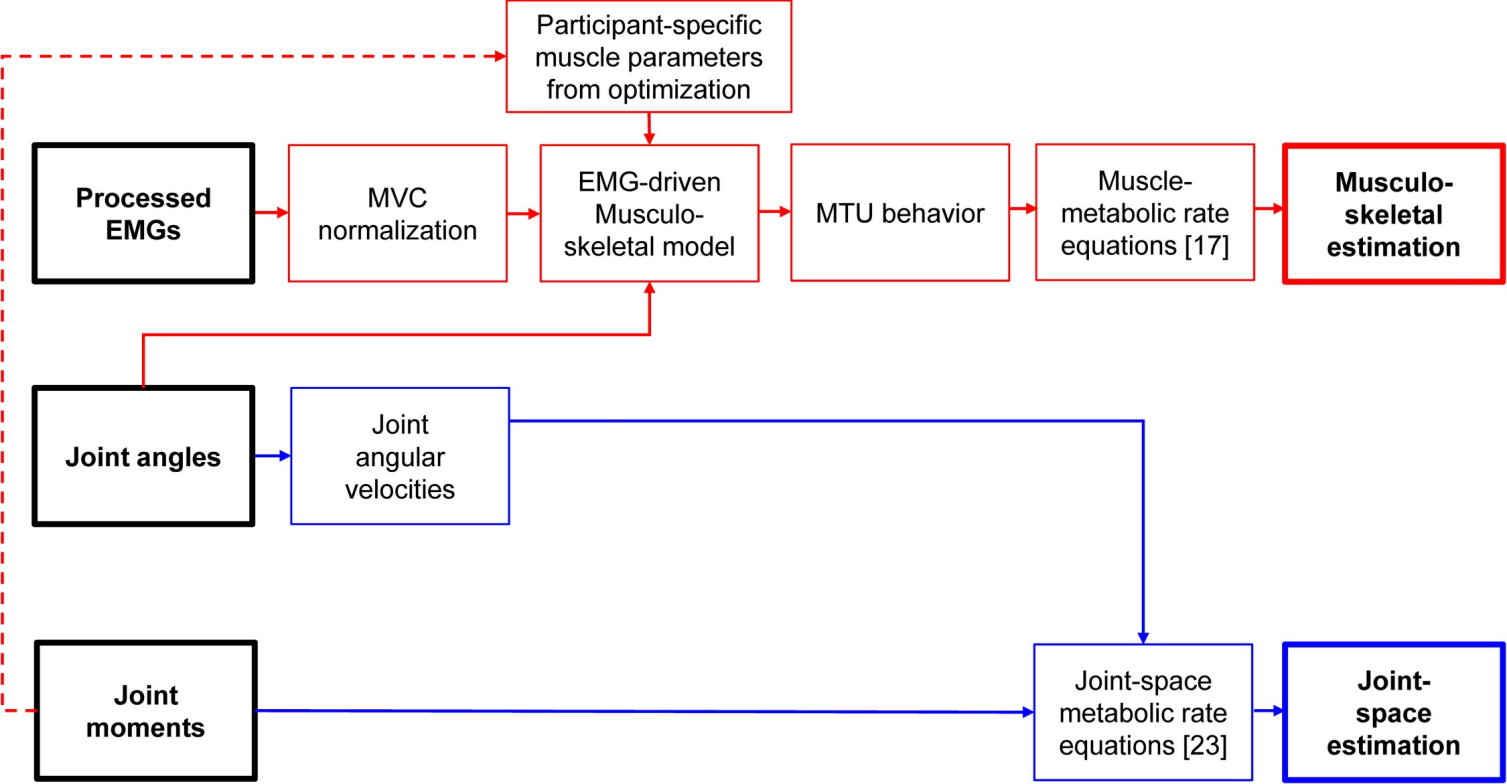
Flow chart of both metabolic rate time profile estimation methods. The musculoskeletal estimation method uses a musculoskeletal model driven by joint kinematics and EMG signals in conjunction with the muscle metabolic cost equations [17]. This method also used joint moment data for optimizing the participant-specific muscle parameters but not for the metabolic rate estimation. The joint-space estimation method uses only the joint kinematics and joint moments as inputs [23].

In the present study, we compared the following two methods for estimating the time profile of metabolic cost: an EMG-driven musculoskeletal simulation with muscle metabolic rate equations from Umberger et al., [17], and a joint-space method [23] (Fig 1). We applied both methods to the same experimental data to exclude the possibility that differences in metabolic rate estimations are due to differences in underlying measurement data. Knowledge about the level of confidence in estimations of the time profile of metabolic cost could be useful for practical applications. For example, in a study that highlights an elastic exoskeleton that reduced the metabolic cost of walking, the authors speculated that one of the conditions with a high spring stiffness reduced the metabolic rate associated with mechanical work during single support, but it increased the metabolic rate associated with muscle activation rate during push-off [33]. Improved metabolic rate time profile estimation methods could be used to verify such hypotheses and to inspire novel assistance strategies to reduce metabolic rate over the entire gait cycle. Improved estimations of the time profile of metabolic cost could also be used to inform which predictive methods realistically simulate walking. Finally, improved knowledge on whether the cost of the swing phase is negligible or not can help justify choices between simulation methods that assume that leg swing is ballistic [34] and simulation methods that include a nonzero leg swing cost [35]. Such knowledge has the potential to advance the study of the energetic determination of human behavior and applications of such methods.

We selected the Umberger metabolic cost equations and the joint-space method because both methods have been introduced specifically for estimating the time profile of metabolic rate in their respective studies [20,23]. Since our goal was not to predict future experimental conditions but instead to use experimental data to estimate a physiological time series (i.e., the time profile of metabolic cost), we used the available EMG data from a recent experiment [36] to drive the musculoskeletal method. This offers the advantage of capturing the neural drive of the participants more realistically than musculoskeletal simulations that estimate the muscle activation through an optimization [37]. We chose to use the Umberger metabolic rate equations since these are currently the most frequently used equations, and because they have proven to correlate well with indirect calorimetry measurements [12]. An earlier version of the joint-space method also showed acceptable correlations in this comparison [12]. The musculoskeletal method and the joint-space method are fundamentally different. In the musculoskeletal method, we used the EMG and kinematics as input, whereas in the joint-space method, we used only joint moments and joint angular velocities (Fig 1). The musculoskeletal method is more tied to theories about the metabolic cost. In contrast, the joint-space method is based on empirical data that was used to estimate relationships between joint behavior and metabolic cost [23].

Hicks et al., [38] propose a validation strategy where estimations are made for multiple movement conditions and the relative change in estimations are compared to the relative change in the reference measurements. We conducted such a trend-validation study by comparing the changes in estimated stride average metabolic rate to the changes from indirect calorimetry measurements during walking at different treadmill grades (downhill, level, and uphill) and walking with different shoe inclinations (downward, level, and upward). The treadmill grade conditions are of interest because they lead to substantial changes in metabolic rate, and the results can be compared to other metabolic rate estimation studies [12,39]. Downhill walking can cause increases in muscle activation [40] required to perform negative joint work [41] and thereby can be used to evaluate how well both methods estimate the changes in metabolic rate due to negative work. Furthermore, walking with different shoe inclinations is of interest since it mimics the effects of suboptimal muscle lengths (e.g., in cerebral palsy patients [8]). As suggested by Koelewijn et al. [12], different walking conditions can serve as a test case of whether estimation methods could be useful in estimating the effects of clinical interventions. Estimations of the time profile of metabolic rate, which is the main focus of the present study, can be improved by scaling the estimated time profile such that the stride average corresponds to the absolute metabolic rate from indirect calorimetry. Therefore, we did not evaluate how accurately the estimation methods tracked changes in absolute units (W kg^-1^). Instead, we focused on how well they tracked changes in relative units (% of reference condition). We evaluated the correlations between changes in costs of different phases of the gait cycle between both estimation methods, and we further compared the estimations to values reported in the literature. There is no direct measurement of the time profile of metabolic rate; thus, this comparison between estimations of the cost of different phases provides an initial view of the variability between estimations from different methods, but it is not an absolute validation.

## Materials and methods

### Ethics statement

The study was approved by the University of Nebraska Medical Center Institutional Review Board. Prior to the experiment all participants provided written consent for data collection, analysis, and publication.

### Experimental dataset

**Fig 2:**
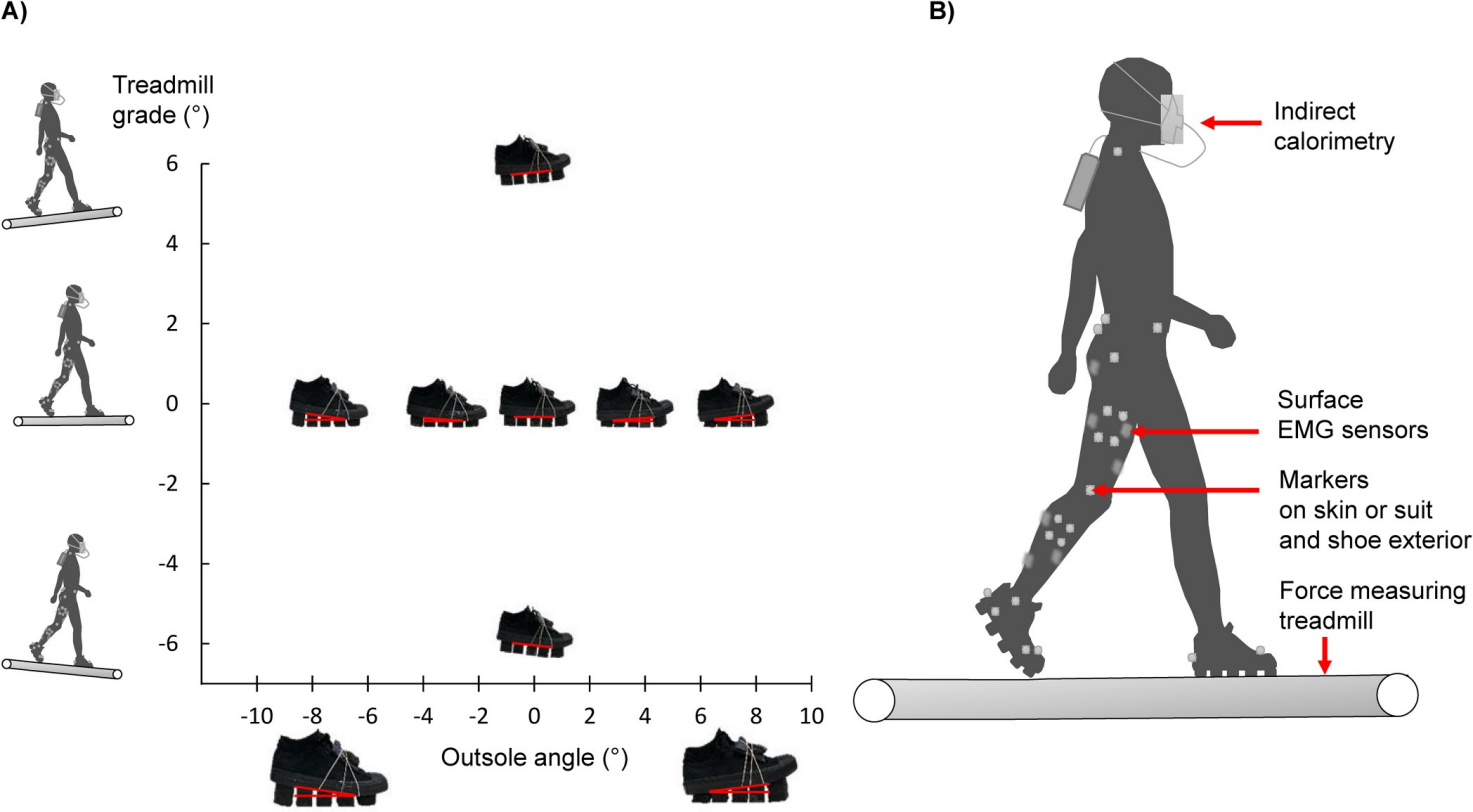
Experimental procedures. **(a)** Conditions. We analyzed previously collected data from walking with level shoes at downhill, level and uphill treadmill grades (-6, 0 and +6°), and walking on a level treadmill with shoes with different outsole inclinations (from -7 to +7°) [36]. The red lines indicate the treadmill grades and shoe inclinations. **(b)** Measurements. We measured metabolic rate using indirect calorimetry, 3D kinematics using motion capture, ground reaction forces using a force treadmill, and muscle activation using surface EMG sensors.

We conducted analyses on data from a previous experiment that involved walking with different types of modular shoes and at different grades during multiple five-minute conditions [36] (Fig 2A). The metabolic rate was recorded using indirect calorimetry (K5, Cosmed, Rome, Italy). We recorded 3D kinematics using motion capture (VICON Vero, Oxford Metrics, Yarnton, UK; 2000 Hz) from 41 reflective markers placed on anatomical landmarks on the skin or tight-fitting suit and on the exterior of the shoes according to a modified Helen Hayes marker set [42]. We recorded ground reaction forces using a treadmill with separate belts that measure the separate ground reaction forces of each leg (Bertec, Columbus, OH, USA; 2000 Hz), and muscle activation using EMG (Trigno TM, Delsys, USA; 2000 Hz). Motion capture and EMG measurements lasted 30 seconds during the last minute of each condition (Fig 2B). Resting metabolic rate was also recorded during a five-minute standing condition before the walking conditions. Since metabolic cost estimation methods require data from all the recorded muscles and joints, we selected data from a subset of 6 participants (age 23.5 ± 2.1 years, mass 79.9 ± 13.8 kg, height 175.7 ± 7.0 cm; mean ± SD) that included trials only where the EMG recordings from all muscles were free from artifacts and had no marker trajectory gaps. We recorded EMG of the muscles of the right leg that represent the different primary functions required for sagittal plane movement during walking: soleus (uni-articular plantarflexion), gastrocnemius medialis (bi-articular plantarflexion), tibialis anterior (dorsiflexion), vastus medialis (uni-articular knee extensor), rectus femoris (knee extension and hip flexion), biceps femoris (knee flexion and hip extension) and gluteus maximus (uni-articular hip extension). The conditions included walking at 1 m∙s^-1^ with level shoes at three treadmill grades (6° downhill, level, and 6° uphill) and walking on a level treadmill with five shoe inclinations (- 7°, - 3°, 0°, 3°, 7°). The treadmill grade conditions are of interest because they lead to large changes in metabolic rate and have been used as a paradigm for evaluating metabolic rate estimation methods [30,39]. The shoe inclination conditions allow us to evaluate whether metabolic time profile estimations are sensitive enough to measure more subtle changes in metabolic cost for the stance phase due to the altered muscle configurations. Additional details on the experimental procedure that were used in the present study can be found in the study by Antonellis et al., [36].

### Data processing

**Fig 3:**
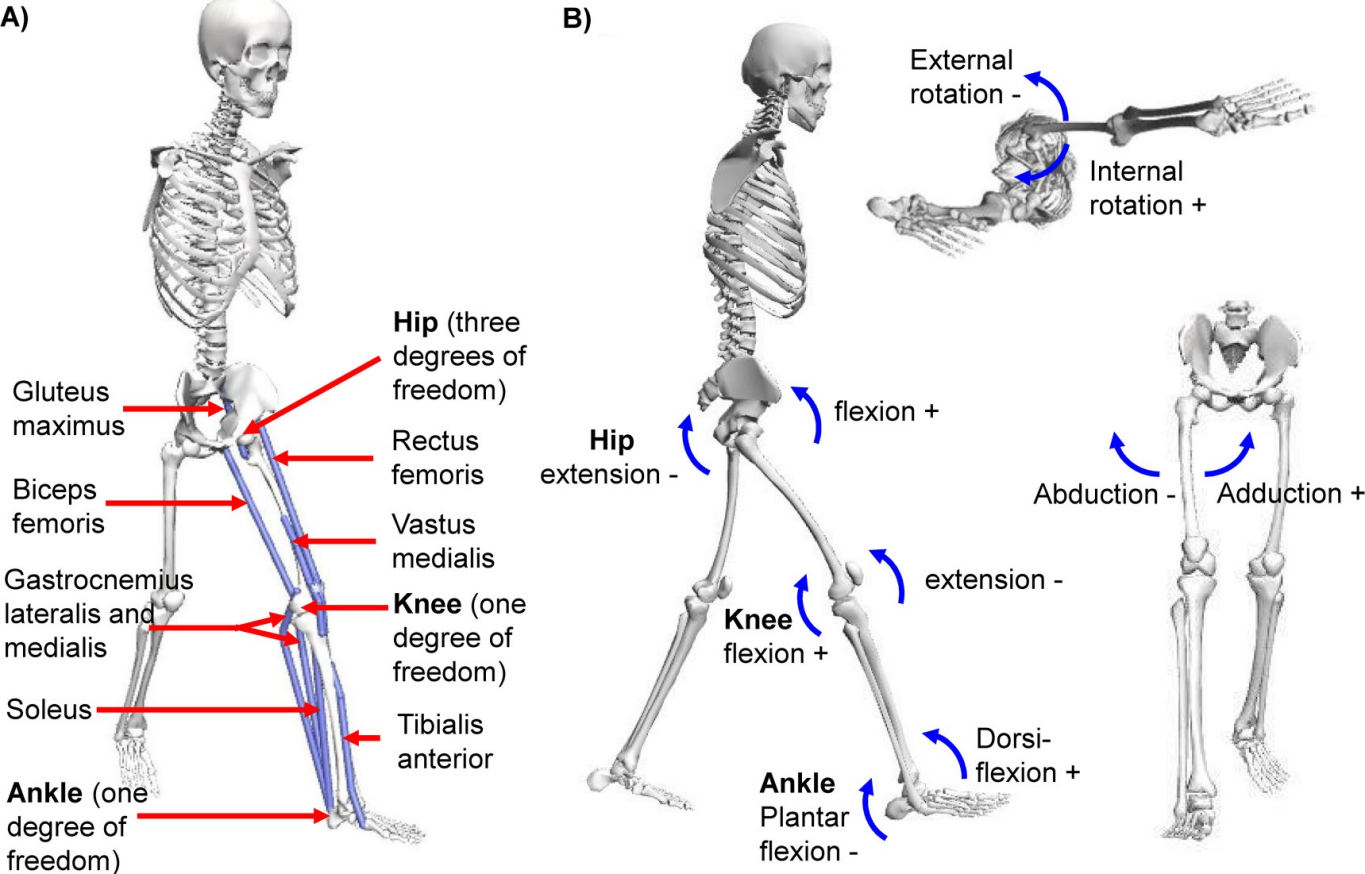
Models for musculoskeletal and joint-space methods. Kinematic, kinetic, and musculoskeletal analyses were performed in OpenSim using a model based on Rajagopal et al., [48]. **(a)** Musculoskeletal method. Schematic of the model that was used to estimate muscle-tendon paths and calculate the muscle metabolic rate, and the degrees of freedom that were used for each joint. The muscles shown were simulated based on EMG recordings. **(b)** Joint-space method. Schematic of the joints that were used to estimate the metabolic rate time profile using the method from Roberts et al., [23] and the sign conventions for joint angular velocity and joint moment.

**Fig 4:**
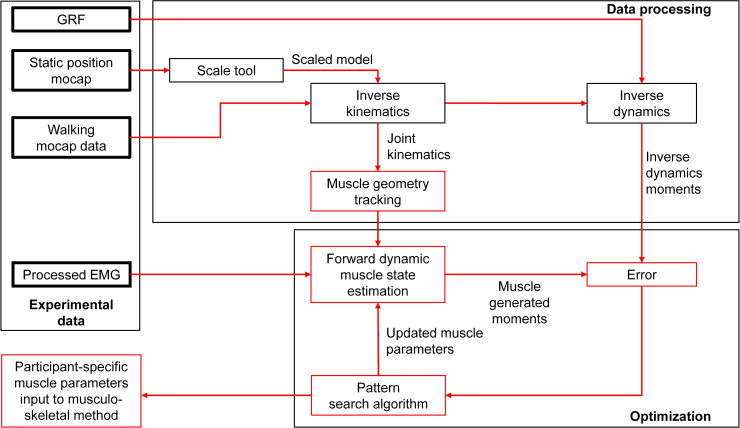
Processing methods. Flowchart describing the steps that were used to process the recorded data for the inverse kinematics, inverse dynamics, and forward dynamics muscle state estimation. The default muscle parameters from the scaling step (tendon slack length and optimal fiber length) were optimized with a generalized pattern search algorithm to maximize the agreement with the moments from the inverse dynamics. The optimized muscle parameters were used as inputs for the musculoskeletal simulation that was used for the estimation of metabolic rate with muscle metabolic rate equations. The data from the kinematic and inverse dynamic analyses were used as inputs for the joint-space metabolic rate estimation.

Breath-by-breath metabolic cost was calculated based on oxygen consumption and carbon dioxide production across the last two minutes of each condition using a standard formula [43]. The net metabolic rate of walking was calculated by subtracting the metabolic rate of standing at rest from that under each walking condition (S1 Table). EMG data were high-pass filtered with a 20 Hz cut-off, then rectified and low pass filtered with a 6 Hz cut-off. Processed EMG signals were normalized based on maximum voluntary contraction (MVC) measurements for each joint. We verified that the magnitudes of the normalized EMG signals were within similar ranges as normative data for EMGs normalized to MVC measurements [44]. In one participant for whom the normalized EMG signals of the soleus and gastrocnemius medialis during the uphill walking trial exceeded the MVC value, we re-normalized those EMGs to the maximum value of this trial [19]. We filtered the ground reaction forces with a low pass Butterworth filter with a 6 Hz cut-off [36,45]. Motion capture data were processed using OpenSim (version 4.0, SimTK, Santa Clara County, California; [46,47]) with the model from Rajagopal et al., [48] (Fig 3A). We chose this model since it is based on detailed morphological data from cadavers and magnetic resonance imaging. The arms were removed from the original model since we did not record their kinematics. We scaled the model using the scaling tool based on motion capture data from a static pose with the “adjust model markers” and “preserve mass distribution” options. The calculated mass distribution was adjusted to account for the mass of the shoes. The inverse kinematics tool was used to estimate the joint kinematics and muscle paths from the marker data (Fig 4). In this procedure, we restricted the ankle and knee motion to one degree of freedom (flexion and extension), which should be a reasonable approximation for walking, but we used all three degrees of freedom for the hip, to realistically simulate the hip movement of walking. We used the inverse dynamics tool with a 6 Hz cut-off filter setting for the marker coordinates to calculate the joint moments. Furthermore, the joint moments were used as inputs for the joint-space estimation method of the metabolic rate time profile [23] (Fig 3B) and as inputs for an optimization of the muscle parameters in the musculoskeletal method. The analysis tool was applied with a 6 Hz cut-off filter to calculate 3D kinematics, which were also used for estimating the metabolic rate with the joint-space method [23] as well as for driving the musculoskeletal simulation. We segmented all data into strides that start at the ipsilateral heel strike and end with the next ipsilateral heel strike based on foot contact detection from ground reaction force data (S1 File).

### Musculoskeletal simulation

We removed all muscles from the original model of Rajagopal et al., [48] except for those from which we recorded EMG signals [18]. Similar to a previous study [49], we drove the lateral gastrocnemius based on EMG data from the medial gastrocnemius since EMG signals were only recorded for the medial gastrocnemius. Muscles were simulated as Hill-type muscles using the Millard muscle model in OpenSim [50]. The standard muscle parameters that were provided with the model were used as the initial settings, similar to other studies [50–53] (Table 1). For the maximum isometric force, we used the sums of muscle groups that were represented by the muscles from which we measured the EMG signals. All length-dependent muscle parameters were initially adjusted for each participant by running the scale tool in OpenSim on the motion capture measurement of the standing position (Fig 4). The software calculates a scale factor for how much the length of each muscle-tendon actuator needs to be adjusted for the model to match the experimental marker positions while maintaining the relative geometry. This scale factor for each muscle is then applied to all length-dependent muscle parameters (e.g., tendon slack length, optimal fiber length). We prescribed the motion from the kinematic analysis to the musculoskeletal model using a MATLAB function [54]. Finally, we used the forward dynamics tool in OpenSim with the prescribed motion, the processed EMG signals as controls, and the default actuator settings to simulate the behavior of the different components of the muscle-tendon unit (e.g., fiber length, tendon force). This is different from studies where forward simulation was used to estimate full-body movement from muscle forces and musculoskeletal dynamics that begin from an initial pose and velocity (e.g., [20]). We used the fifth order Runge-Kutta-Merson integrator in OpenSim to solve the dynamic equations for the muscle states in the forward dynamics tool. We did not specify an initial state, which makes the software start from the default muscle states as the initial states.

**Table 1:** Muscle parameters in musculoskeletal simulation and muscle metabolic rate estimation.

	Maximum isometric force (N) [52]	Optimal fiber length (m) [53]	Tendon slack length (m) [53]	Pennation angle at L_opt._ (rad.) [50]	Maximum contraction velocity (L_opt_ s^-1^) [51]	Slow-twitch ratio (%) [55]	Muscle width coefficient [20]
Tibialis anterior	1227	0.07	0.24	0.20	10	70.0	0.49
Soleus	6195	0.04	0.28	0.38	10	80.3	0.80
Gastrocnemius medialis	3116	0.05	0.40	0.17	10	56.6	0.61
Gastrocnemius lateralis	1575	0.06	0.38	0.21	10	50.7	0.61
Vastus medialis	9594	0.10	0.20	0.42	10	50.3	0.76
Rectus femoris	2192	0.08	0.45	0.22	10	38.7	0.76
Biceps femoris	1870	0.10	0.33	0.18	10	54.2	0.78
Gluteus maximus	3338	0.15	0.05	0.35	10	55.0	0.77

L_opt_ is optimal fiber length.

### Muscle-parameter optimization and validation

Different studies use optimization methods to improve the scaling of the muscle-parameters to maximize the agreement between muscle-generated and inverse dynamics moments [19,56–58]. We chose to optimize the tendon slack length and optimal fiber length for each muscle of each participant (Fig 4) since it is expected that joint moments are most sensitive to these two parameters [56,59–62] and several studies that involved an optimization step had used these muscle-parameters [19,56,61]. Our optimization cost function was based on the mean squared errors (MSEs) between the net sagittal plane joint moments generated by the simulated muscles and the net sagittal plane joint moments measured through inverse dynamics. The muscle-generated net joint moments were calculated by multiplying the tendon force of each muscle by its moment arm and by calculating the net result from all the extensors and flexors around each joint. For the hip joint, we only evaluated the mean squared error of the phase where the hip extension moment from inverse dynamics is in the sagittal plane extension direction since our model did not include uni-articular hip extensor muscles. Hip flexion during walking appears to come mostly from stretch and recoil of ligaments instead of muscles [19,63], and not modeling metabolic cost from uni-articular hip flexors, therefore, appears to be a reasonable approximation. In the cost function, we included a number of penalty terms that, through trial-and-error, were found to be useful to avoid unrealistic solutions [20,64,65]. One term was designed to avoid solutions where a muscle did not apply force. Another penalty term was designed to avoid solutions where the tendon force of the soleus was lower than the mean tendon force of the medial and lateral gastrocnemii [66,67]. Similar to Markowitz and Herr, [19], we also used a penalty term to constrain tendon slack lengths and optimal fiber lengths to stay within physiologically realistic operating conditions. In summary, the cost function was as follows:

$$Cost\;function=MSE_{ankle}+MSE_{knee}+MSE_{hip\;extension}+c_{1}\cdot ZeroForcePenalty+c_{2}\cdot PlantarflexorDistributionPenalty+c_{3}\cdot PhysiologicalRangePenalty$$ (1)

where *c*_*1*_, *c*_*2*_, and *c*_*3*_ were all set to 10^6^. These coefficients were chosen to be much larger than the mean squared errors, such that the optimization quickly moves to regions where all the penalty terms are zero. *ZeroForcePenalty* is equal to the number of muscles for which the tendon force stays zero over the entire stride. *ZeroForcePenalty* is zero if all the tendon forces are greater than zero for at least a part of the stride. *PlantarflexorDistributionPenalty* is equal to one if the tendon force of the soleus is lower than the mean tendon force of the medial and lateral gastrocnemii. Otherwise, *PlantarflexorDistributionPenalty* is zero. *PhysiologicalRangePenalty* is equal to the number of muscles for which the following boundary criteria are not satisfied:

Fiberlength≥optimalfiberlength·(1−width) (2)

and

fiberlength≤optimalfiberlength·(1+width) (3)

where *fiber length* is the adjusted fiber length of each simulation iteration, *optimal fiber length* is the adjusted optimal fiber length of each simulation iteration, and width is the width of the force-length curve [20]. If the fiber length stays between the boundaries for all muscles, then *PhysiologicalRangePenalty* is zero.

For each participant and muscle group, we searched for the tendon slack lengths and the optimal fiber lengths that minimized the cost function using the generalized pattern search function in MATLAB. The algorithm was programmed to identify the optimal muscle parameters starting from the initial output from the scale tool, within a range from 50 to 150% [56]. The algorithm terminated when all the penalty terms were zero, and the improvements in the cost function were smaller than 10^−6^ or 300 iterations had been completed. The optimized muscle parameters (Table 2) were used in the simulations for the metabolic rate estimations. To evaluate the final result of the optimization, we reported the root mean square error of the muscle-generated moments compared to the joint moments from inverse dynamics.

**Table 2:** Optimized muscle parameters. Optimal fiber lengths and tendon slack lengths that minimize the cost function and were used for calibrating the muscle parameters (mean ± inter-participant s.e.m.).

	Optimal fiber length (m)	Tendon slack length (m)
**Tibialis anterior**	0.06 ± 0.01	0.25 ± 0.01
**Soleus**	0.06 ± 0.01	0.29 ± 0.01
**Gastrocnemius medialis**	0.08 ± 0.01	0.44 ± 0.01
**Gastrocnemius lateralis**	0.09 ± 0.01	0.42 ± 0.01
**Vastus medialis**	0.12 ± 0.01	0.21 ± 0.01
**Rectus femoris**	0.09 ± 0.01	0.45 ± 0.01
**Biceps femoris**	0.13 ± 0.02	0.35 ± 0.02
**Gluteus maximus**	0.14 ± 0.00	0.02 ± 0.00

### Musculoskeletal metabolic rate estimation

Metabolic rate was estimated from the musculoskeletal simulation using a version of muscle metabolic rate model [17] with additional modifications [16,20,68]. The muscle density in the simulation is set to 1059.7 kg m^-3^ [69] (Table 3). The ratio of isometric force divided by the physiological cross-sectional area (i.e., specific tension) is set to 600,000 N m^-2^ [48] and is used to obtain the muscle masses. We used the default slow-twitch ratios in OpenSim [55]. Metabolic energy consumption is the result of muscle fiber mechanical work and energy loss from heat production. Consequently, the metabolic cost equations partitioned metabolic rate into fiber mechanical work and two heat rates: activation-maintenance heat rate and shortening-lengthening heat rate. A scaling factor that depends on whether the effort is primarily aerobic or anaerobic in nature (i.e., aerobic factor) was set to the default value for aerobic activities of 1.5 [17,70]. We used the orderly fiber recruitment model from Bhargava et al., [10]. Mechanical work adsorption from muscle fibers (i.e., negative mechanical work) was included, as proposed in the original model from Umberger et al., [17], and implemented in recent simulation studies [18]. The total metabolic rate per muscle was enforced to be no less than zero based on the concept that negative mechanical work cannot lead to ATP synthesis [14].

**Table 3:** Estimated metabolic rate (% mean ± s.e.m.) of different phases of the gait cycle during level walking from the current work and other studies. We segmented the strides into double support phases, single support phase, and swing phase based on our ground reaction force data from both legs. Strides are segmented from the ipsilateral heel strike to the next ipsilateral heel strike such that the first double support phase starts with an ipsilateral heel strike and ends with contralateral toe-off, and the second double support phase starts with contralateral heel strike and ends with ipsilateral toe-off. The metabolic rate time profile in the normal walking condition from the Jackson et al., [18] study was obtained by applying the modified Umberger [20] estimation code from [68]. The data from Roberts et al., [23] were obtained from their supplementary data file for walking at 70% of the preferred walking speed. We selected this trial from their supplementary data because the walking speed is most similar to the walking speed from our study (1 m∙s^-1^). We obtained the unilateral metabolic rate from the right ankle knee and hip joints by entering the data from the right ankle knee and hip as inputs to their MATLAB code and entering signals with zeros for all non-lower limb joints and all joints on the left side of the body.

	First double support (1 to 15% of stride) (%)	Single support (15 to 50% of stride) (%)	Second double support (51 to 65% of stride) (%)	Swing (66 to 100% of stride) (%)	Sum of double support phases (%)
**Own musculo-skeletal estimation**	17 ± 1.3	41 ± 2.7	19 ± 4.0	22 ± 1.8	37 ± 3.0
**Own joint-space estimation**	10 ± 0.7	26 ± 2.3	49 ± 2.7	15 ± 1.0	60 ± 2.8
**Jackson et al.,** [18]	10 ± 0.5	39 ± 2.8	27 ± 3.0	24 ± 1.9	37 ± 2.8
**Roberts et al.,** [23]	10	28	39	23	49
**Umberger (calculations based on digitized time profile)**	27	40	8	25	35
**Markowitz and Herr,** [19]	N/A	47	N/A	26	27
**Grabowski et al.,** [26]	N/A	55	N/A	N/A	45
**Griffin et al.,** [27]	99–76			1–24	N/A
**Gottschall and Kram.,** [29]	90			10	N/A
**Doke et al.,** [28]	83			17	N/A

### Joint-space metabolic rate estimation

Joint angular velocities and joint moments were used as inputs. The MATLAB script from Roberts et al., [23] was modified to calculate metabolic rate from only the lower limb joints and report metabolic rate from the right side of the body by entering values of zero for the angular velocities and moments of the joints on the other side. We reasoned that reporting the time profile of the metabolic rate of one side of the body for each phase of the gait cycle is more informative than reporting the bilateral average. For example, a plot of the bilateral sum of the time profiles of both sides of the body does not allow us to distinguish which part of the metabolic rate can be attributed to the first and second double support phase. Our modified MATLAB code estimated the joint-space activation-maintenance and shortening-lengthening heat rates and mechanical power that were used towards obtaining the joint-space estimations of metabolic rate.

### Simulation validation

To validate the accuracy of the musculoskeletal simulation, we verified if the muscle-generated moments from the optimized simulation stayed within two times of the standard deviation of the moments from the inverse dynamics as suggested in validation guidelines from Hicks et al., [38]. Additionally, we calculated the root mean squares of the errors between muscle-generated and inverse dynamics moments. Muscle fiber length and force time series were compared to time series from in vivo imaging and force sensing studies for muscles for which literature data was available [71–74]. To verify how similar our estimations are to the literature, we plotted all the EMG, muscle fiber, and joint parameters and metabolic cost components of each muscle and joint versus available supplementary data from the literature (S1 and S2 Figs). The musculoskeletal estimation results were plotted versus the dataset from Jackson et al., [18], which used a similar EMG-driven musculoskeletal simulation approach. The metabolic cost estimation code based on Umberger et al., [17] with modifications from Uchida was used to obtain the heat rates from that dataset [68]. For the joint-space estimation method, we plotted the angular velocity, moment, and estimated metabolic rate of each joint versus data from one of the trials that were published in their supplementary data [23] with the speed that was most similar to our experiment.

### Analysis

Since the musculoskeletal estimation method was based on only eight muscles and the joint-space estimation did not include the angular velocities and moments of the upper body, we did not expect the estimated metabolic rates to match the absolute value of the measured metabolic rate. As such, we evaluated the relative changes in percent. The estimations were converted to percentages of the stride average under the baseline condition (walking with level shoes on a level grade). It is known that indirect calorimetry measurements and biomechanical measurements that affect the estimation methods (EMG) can sometimes have high variability due to measurement noise. Before the analysis, we eliminated data with extreme outliers in stride-average metabolic rates that fell outside a range of the median plus-minus three times the interquartile range [75]. The estimations were performed for all 42 conditions except for one downward shoe condition due to faulty signals from the EMG sensors.

To evaluate how well both methods estimated the relative changes in stride-average metabolic rate, we conducted a repeated measures correlation (R_rm_) analysis [76] of the percent change of the estimation compared to indirect calorimetry. This statistical test takes into account that there is within- and between-subject variation in the data. The tests provide a correlation coefficient and P-value that expresses how strongly and consistently the estimation correlates with indirect calorimetry. Moreover, the repeated measures correlation test provides a slope coefficient that describes whether the estimation underestimates or overestimates the indirect calorimetry measurement depending on whether the slope is smaller or greater than one, respectively. To evaluate the mutual consistency of the time profiles of both estimation methods, we used the repeated measures correlation to compare the two estimation methods on the averages of the different phases of the gait cycle (first and second double support, single support, and swing phase) during each walking condition. A Bonferroni correction was used to adjust for comparisons of multiple conditions. All statistical analyses were performed in MATLAB (MathWorks, Natick, MA, USA), and the ANCOVA function was used to conduct the repeated measures correlation analysis [76]. For all the statistical tests, we set the significance threshold at 0.05.

## Results

### Simulation validation: Muscle-generated moments approximate inverse dynamics

**Fig 5:**
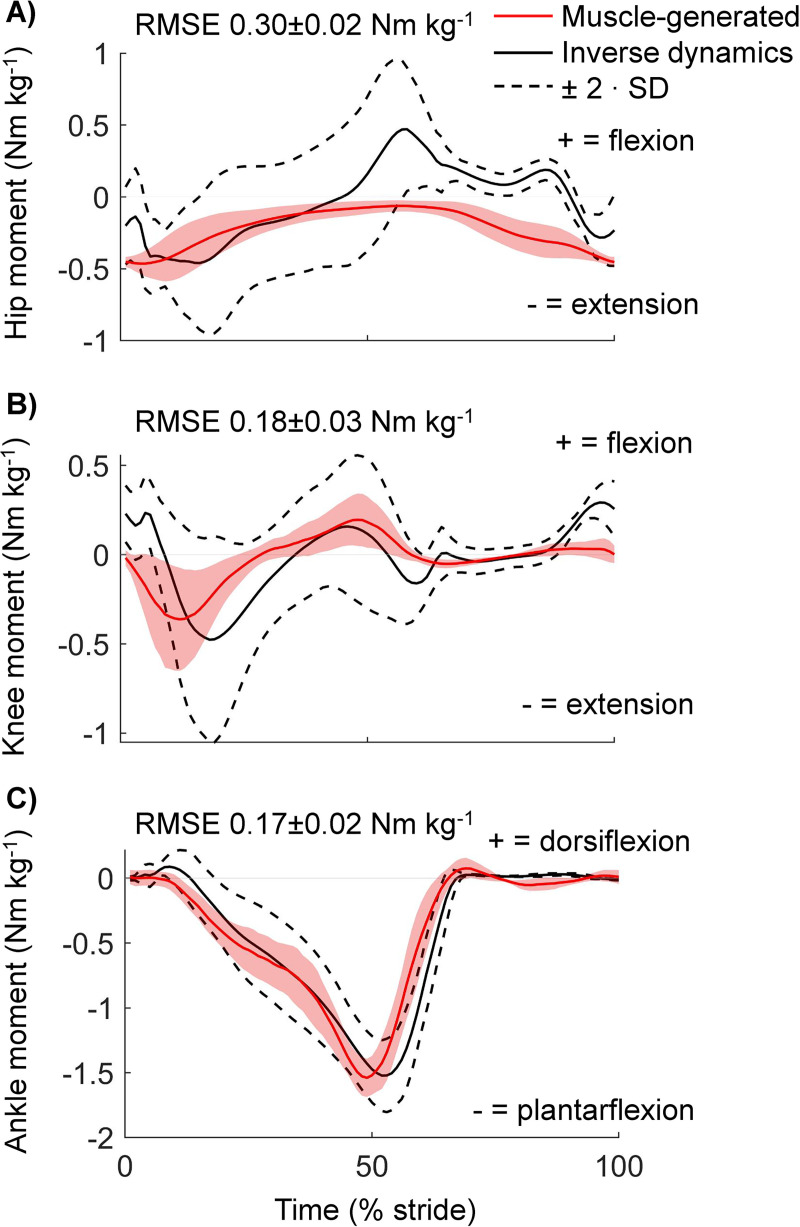
Musculoskeletal method validation. **(a)** Hip moments, **(b)** Knee moments, **(c)** Ankle moments. Red lines represent the net joint moments from the simulated muscles in the normal walking condition. The shaded area is s.e.m. The black line represents the net joint moments from inverse dynamic analyses. Dashed lines represent the mean inverse dynamics joint moments ± two times the standard deviation, which is suggested as a validation threshold [38]. Values in the plots represent mean ± inter-subject s.e.m. of root mean square errors of the difference between muscle-generated and inverse dynamics moments. The muscle-generated hip moments followed the inverse dynamics moments in the extension direction but not in the flexion direction since the optimization algorithm was programmed only to minimize the error during hip extension moment generation.

The time profile of metabolic rate was estimated with an EMG-driven musculoskeletal method and a joint-space method that used joint moments from inverse dynamics analyses. To evaluate the validity of the musculoskeletal simulation, we compared the muscle-generated joint moments with the joint moments from inverse dynamics analysis. The mean muscle-generated moments of the ankle and knee stayed within a range of two times the standard deviation from the inverse dynamics moments for the majority of the stride cycle and had root mean square errors of 0.17 ± 0.02 and 0.19 ± 0.03 Nm kg^-1^ (Fig 5). The muscle generated moment of the hip had a higher root mean square error of 0.30 ± 0.02 Nm kg^-1^, mainly because the muscle-generated moments did not track the hip flexion portion of the inverse dynamics moment. This result was expected because we programmed the optimization algorithm only to minimize the error from the portion of the stride cycle where the inverse dynamics moments are in the extension direction. We chose this option as it is challenging to record surface EMG of the primary hip flexor muscles, and literature suggests that hip flexion during walking mostly comes from tendon stretch instead of muscle contraction [77]. Overall, the errors appear of a similar magnitude as in other metabolic cost estimation studies that evaluate muscle-generated moments [18,19].

### Effects of conditions: Treadmill grade affects total leg work rate, and shoe inclination affects metabolic rate at the ankle

Less downhill and more uphill grades resulted in increasing magnitudes of positive work of the entire leg (i.e., individual leg COM power), average plantarflexion moment, average EMG of the soleus, gastrocnemius medialis and tibialis anterior, and decreasing magnitudes of negative work of the entire leg [36]. The muscle analysis suggests that more uphill grades led to increasing metabolic rate during the entire stance phase, primarily at the ankle followed by the hip and knee, increasing activation-maintenance heat rate and positive fiber work, and decreasing shortening-lengthening heat rate and negative fiber work (S3 Fig). Less downward and more upward shoe inclinations lead to increasing magnitudes of average plantarflexion moment, positive and negative ankle work, and average tibialis anterior EMG. The muscle analysis suggests that downward shoe inclinations led to increasing metabolic rate at the ankle due to increased fiber work during the second double support phase (i.e., from contralateral heel strike to ipsilateral toe-off). Upward shoe inclinations led to increasing metabolic rate at the ankle, which is related to an earlier increase in activation-maintenance heat rate during single support (S4C and S4E Fig). More detailed information about the effects of the conditions on joint moments, powers, and muscle activations are provided in the study that reports the experimental dataset [36].

### Stride-average metabolic rate: Estimations correlate with large indirect calorimetry changes from grade walking but not subtle changes from footwear

**Fig 6:**
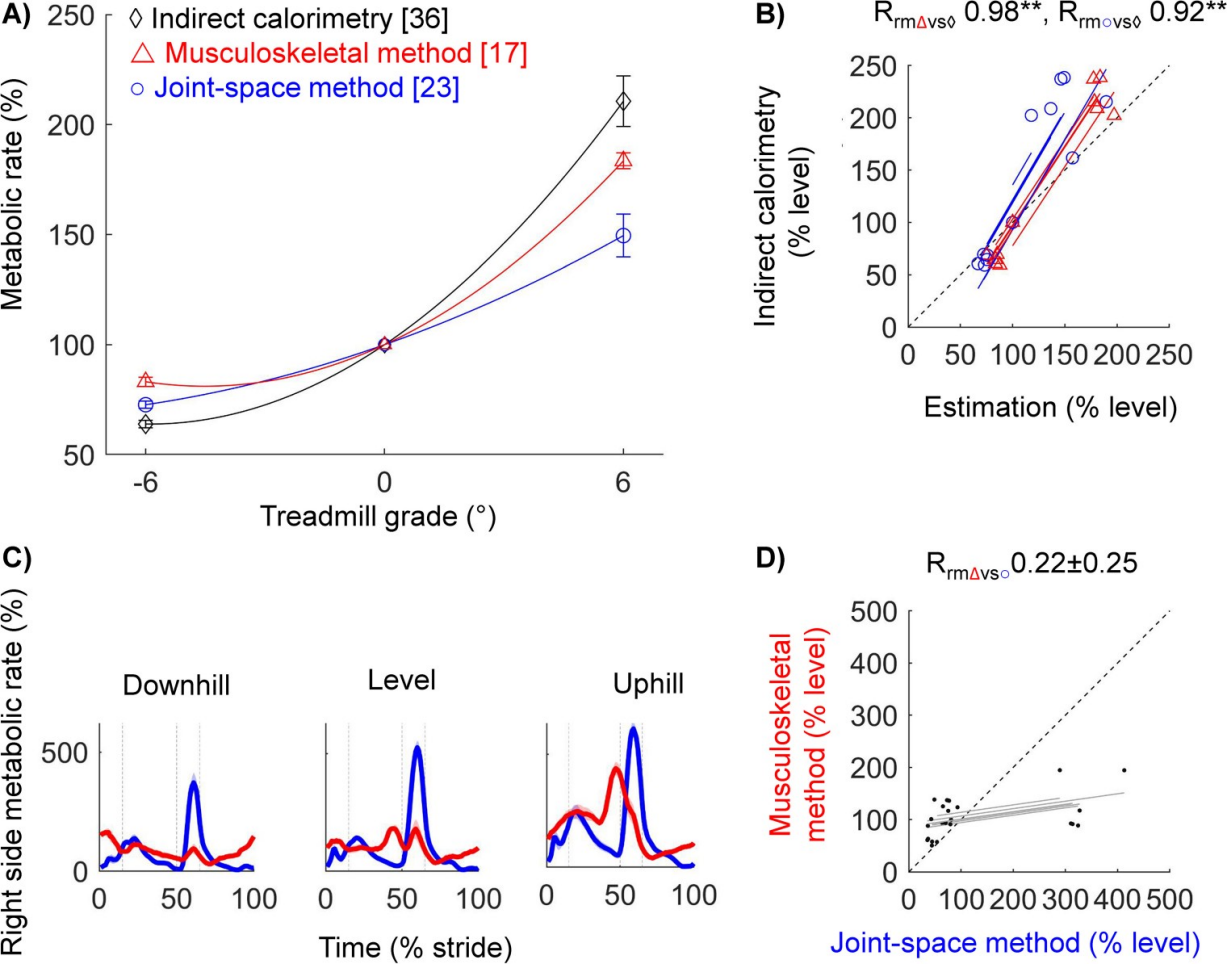
Estimations of the stride average metabolic rate and the time profile of metabolic rate during downhill, level, and uphill walking with level shoes. **(a)** Stride average metabolic rate. Red triangles, blue circles, and black diamonds indicate the results from the musculoskeletal estimation, the joint-space estimation method, and indirect calorimetry, respectively. Error bars indicate s.e.m. The average trends across conditions from the same method are shown as a solid line from a second-order polynomial curve fit. **(b)** Individual stride averages. Symbols are individual trials. Lines are individual fits from the repeated measures correlation test. Slopes that are less or more steep than a slope of 1/1 indicate overestimation or underestimation, respectively. R_rm_-values indicate the repeated measures correlation. ** indicate that the P-value of the repeated measures correlation is ≤ 0.01. **(c)** Time profiles under different treadmill conditions. Red and blue lines indicate the results from the musculoskeletal estimation and joint-space estimation methods, respectively. Strides are segmented from the ipsilateral heel strike to the next ipsilateral heel strike. The shaded area indicates s.e.m. **(d)** Individual phase averages. Symbols are averages of the phases during the level walking condition separated by vertical lines: first double support (from ipsilateral heel strike to contralateral toe-off), single support, second double support (from contralateral heel strike to ipsilateral toe-off), and swing phase. R_rm_-values indicate mean ± s.e.m. of the repeated measures correlations of the different walking conditions.

**Fig 7:**
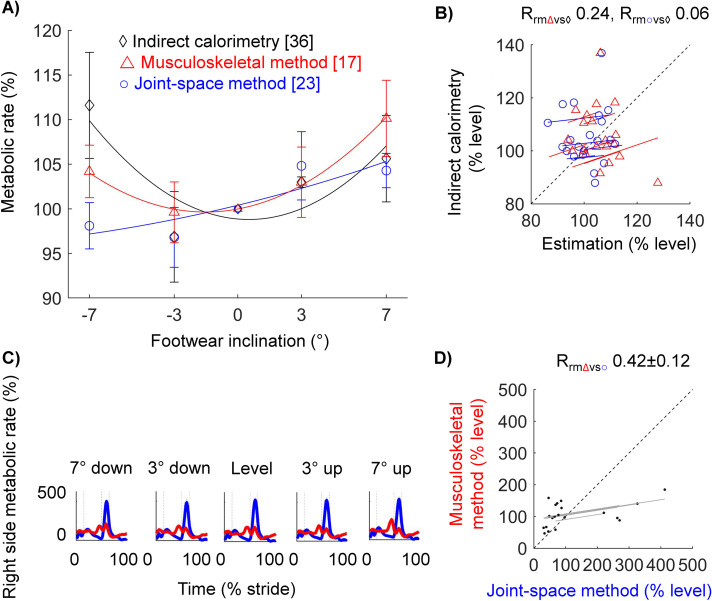
Estimations of the stride average metabolic rate and the time profile of metabolic rate during walking on a level grade with different shoe inclinations. **(a)** Stride average metabolic rate. Red triangles, blue circles, and black diamonds indicate the results from the musculoskeletal estimation method, the joint-space estimation method, and indirect calorimetry, respectively. Error bars indicate s.e.m. The average trends across conditions from the same method are shown as a solid line from a second-order polynomial curve fit. **(b)** Individual stride averages. Symbols are individual trials. Lines are individual fits from the repeated measures correlation test. Slopes that are less or more steep than a slope of 1/1 indicate overestimation or underestimation, respectively. R_rm_-values indicate the repeated measures correlation. **(c)** Time profiles under different shoe inclination conditions. Red and blue lines indicate the results from the musculoskeletal estimation and joint-space estimation methods, respectively. Strides are segmented from the ipsilateral heel strike to the next ipsilateral heel strike. The shaded area indicates s.e.m. **(d)** Individual phase averages. Symbols are averages of the phases during the level walking condition separated by vertical lines: first double support (from ipsilateral heel strike to contralateral toe-off), single support, second double support (from contralateral heel strike to ipsilateral toe-off), and swing phase. R_rm_-values indicate mean ± s.e.m. of the repeated measures correlations of the different walking conditions.

To investigate the accuracy of both methods in estimating the changes in the stride-average metabolic rate, we compared the estimations of both methods to changes in average metabolic rate measured with indirect calorimetry. Compared to the metabolic rate for walking on a level treadmill, the metabolic rate for downhill walking on a 6° grade was reduced by 17 ± 2, 27 ± 2, and 36 ± 2% (mean ± standard error of the mean [s.e.m.]), and the metabolic rate for uphill walking on a 6° grade increased by 83 ± 4, 49 ± 10, and 111 ± 12% using estimations from the musculoskeletal simulation method, the joint-space method, and indirect calorimetry, respectively (Fig 6A, S2 Table). For walking on a level treadmill with shoes with a 7° downward inclination, we estimated increases in metabolic rate of 4 ± 3 and 12 ± 6% compared to that for walking with level shoes using the musculoskeletal estimation method and indirect calorimetry, respectively (Fig 7A). In contrast, the joint-space method estimated a reduction in metabolic rate of 2 ± 2%. For walking with shoes with a 7° upward inclination, we estimated increases in metabolic rate of 10 ± 4, 4 ± 2, and 6 ± 5% using the musculoskeletal estimation method, the joint-space estimation method, and indirect calorimetry, respectively.

The estimations of the stride average metabolic rate from the musculoskeletal and joint-space estimation method showed strong positive correlations with changes in indirect calorimetry measurements from walking on different grades (R_rm_ = 0.98, P < 0.01 for musculoskeletal method versus indirect calorimetry; R_rm_ = 0.92, P < 0.01 for joint-space method versus indirect calorimetry; Fig 6B). Indirect calorimetry changes followed a slope greater than one for both methods, which indicates that both methods underestimated indirect calorimetry measurements (slopes were 1.51 and 1.72 respectively for the musculoskeletal method and joint-space method versus indirect calorimetry). Correlations of both estimation methods versus the subtle changes in indirect calorimetry from walking with different shoe inclinations were weak and not significant (R_rm_ of musculoskeletal method versus indirect calorimetry is 0.24, P = 0.29; R_rm_ of joint-space method versus indirect calorimetry is 0.06, P = 0.80, Fig 7B). Inspection of the spatial distribution of the average metabolic rate over different muscle groups reveals that the musculoskeletal method resulted in the largest estimation of the metabolic rate for the plantarflexors (57 ± 3.1% of stride average, Table 4), followed by the knee extensors and hip extensors, and with the lowest estimation for the knee flexors and dorsiflexors (both 5 ± 1.4%). According to the joint-space method, the highest portion of metabolic cost also came from the plantarflexors (41 ± 3.5%) and the lowest portion also came from the dorsiflexors (1 ± 0.2%). However, the order of the muscle groups in between was slightly different with the second and third highest portions for the hip extensors and hip flexors followed by the knee extensors and knee flexors.

### Metabolic rate time profile estimations show moderate to weak correlations

To evaluate the mutual agreement between the metabolic rate time profile estimations of the musculoskeletal method and the joint-space method, we evaluated the correlation in the estimations of metabolic rates of different phases. According to the musculoskeletal method, the majority of the metabolic cost of level walking comes from the single support phase (41 ± 3%), followed by from the swing phase (22 ± 2%), the second double support phase (19 ± 4%, from contralateral heel strike to ipsilateral toe-off), and the first double support phase (17 ± 1%, from ipsilateral heel strike to contralateral toe-off) has the lowest metabolic cost (Table 3, Fig 6C, Fig 7C). According to the joint-space method, the majority of the metabolic cost of level walking does not come from the single support phase but the second double-support phase (49 ± 3%), followed by the single support phase (26 ± 2%), the swing phase (15 ± 1%) and the first single support phase also comes last, similar to the musculoskeletal method (10 ± 1%). Estimations of the metabolic cost of different phases showed weak positive correlations between both methods that were not significant, except for the most downward shoe inclination (R_rm_ = 0.67, P = 0.020 for 7° downward shoe condition, R_rm_ = 0.35 ± 0.16, P ≥ 0.243 for all other shoe conditions, Fig 7D; R_rm_ = 0.22 ± 0.25 and P ≥ 0.146 for all treadmill grades, Fig 6D). The slopes of estimations of the costs of phases from the musculoskeletal method versus the joint-space method were smaller than one, which indicates that the musculoskeletal method estimated smaller fluctuations in metabolic rate within the stride cycle than the joint-space method.

## Discussion

The aim of this study was to evaluate two methods that allow for the estimation of the time profile of metabolic cost within the gait cycle within the same dataset. The time profiles of the musculoskeletal and joint-space methods showed weak positive correlations with each other that were mostly not significant, which highlights that both methods provide different time profile estimations. Both estimations of stride average metabolic rate were sufficiently accurate to correlate significantly with changes in indirect calorimetry measurements from walking on different grades, but not from walking with different shoe inclinations. Since it is not possible to validate the time profile estimations against direct measurements of metabolic rate time profiles, these evaluations of stride-average estimations can provide insights into the potential accuracy: If a method could accurately estimate the time profile of metabolic rate, it would also likely be able to estimate changes in stride average metabolic rate. Our results suggest that both methods were accurate enough to estimate large but not subtle changes in metabolic rate, such as changes from shoe inclinations. The correlations of changes in stride average metabolic rate versus indirect calorimetry were slightly higher for the musculoskeletal method than for the joint-space method. Compared to the musculoskeletal method, the joint-space estimation method could be expected to have a weaker correlation to indirect calorimetry because it was developed based on a dataset from walking at different speeds [23] and not from walking at different grades or with different types of shoes [36]. The changes from the footwear conditions were not always estimated in the correct direction, possibly due to counterintuitive effects on muscle-tendon dynamics, which are not always captured in joint biomechanics. The joint-space method might not be sufficiently accurate for estimating the effects of devices with complex interactions, such as with exoskeletons.

The first double support phase had the lowest metabolic cost according to both estimation methods (respectively 17 and 10% according to the musculoskeletal and joint-space method), but according to the musculoskeletal method, the single support phase was the phase with the highest metabolic cost (41%) whereas the joint-space method estimated the highest metabolic cost during the second double support phase (49%, Table 3). While there are no direct measurements of the time profile of metabolic rate, our results can be compared to estimates from the literature. Our estimations of the relative cost during the swing phase (~22% according to the musculoskeletal method and 15% according to the joint-space method) are within the range from studies with experimental perturbations (1 to 24% based on added mass experiments [27]; 17% based on experiments with isolated leg swinging [28] and 10% based on an experiment with elastic swing leg assistance [29]). Our musculoskeletal estimation of the total cost of the double support phases is close to the estimation based on added mass and body weight support experiments (37% in our data versus 45% in [26]). We also compared our data to the results for individual muscles and joints from supplementary datasets from previous musculoskeletal and joint-space estimation studies. We found that the costs of different phases (first and second double support, single support, and swing phases) from our musculoskeletal method were less than 10% of the mean values derived from the normal walking condition data in the study from Jackson et al., [18] (Table 3, S1 and S2 Figs). This agreement with the results from Jackson et al., [18] makes sense because our study also used an EMG-driven approach. The main differences of our study compared to Jackson et al., [18] are that we did not rely on optimization to scale the EMG measurements since we had MVC measurements and that we included a hip extensor muscle. The metabolic rates of the different phases from the joint-space estimations are also within 10% of values derived from data from the Roberts et al., [23] study, which used similar methods to the joint-space estimation in our study. One main difference of our analysis compared to their study is that we did not have motion capture data of the joints in the upper body. Therefore, we did not include contributions of the upper body joints to metabolic rate.

The costs of phases appear to be similar to those from studies that used similar methods as our study (when comparing the musculoskeletal estimation method to data derived from [18] and when comparing the joint-space estimation to data derived from [23]). However, our estimations of the cost of the second double support phase are more than two times greater than that of Umberger (respectively 19 and 49% with the musculoskeletal and joint-space methods, compared to only 8% in Umberger [20]). Our estimations of the cost of the sum of the double support phases are greater than that of Markowitz and Herr (respectively 37 and 60% with the musculoskeletal and joint-space method compared to 27% in Markowitz and Herr, [19]). While we do not know the exact causes of these differences, it is known that the choice of methods to estimate the change of Hill-type muscles and metabolic-rate equations can have vast influences on the resulting estimations [12,14]. The study from Umberger [20] used forward simulation to predict the kinematics and muscle behavior of a 2D model with 12 muscles per side. The metabolic rate was then estimated with the equations from Umberger et al. [17], with the modification where the metabolic rate from negative work was not included. Markowitz and Herr, [19] used joint kinematic measurements and EMG measurements (supplemented by EMG measurements of deeper muscle layers from the literature) to drive a 2D model with 14 muscles per leg in a similar EMG-driven method as our study. One major difference compared to our study is that they used a multi-objective optimization to optimize muscle parameters (e.g., tendon slack length) for minimizing metabolic rate estimated with the original equations from Umberger et al., [17] and maximizing the correlation between estimated and measured joint moments. We chose to use recent modifications that are designed to represent the human body more realistically, such as a 3D model [48] with muscles that wrap over curved surfaces (e.g., epicondyles) instead of 2D models with straight-line muscles [19,20]. To realistically capture the neural drive, we used EMG measurements to drive the simulation instead of estimating the activation through optimization [20], and we used the updated muscle metabolic cost equations [16] rather than the original equations [17,19]. These methodological differences could explain the different results in our study compared to the studies from Umberger [20] and Markowitz and Herr, [19].

If we had conducted an experiment with a hip exoskeleton [78] or an ankle tether that pulls on the leg during the swing phase [29], it could be expected that effects on metabolic rate can exist during the swing phase. In contrast, the inclined treadmill and inclined shoe conditions do not apply any forces to the leg during the swing phase. Therefore, we expected that most of the effects of the conditions on the metabolic rate would likely occur during the stance phase. The results of both metabolic time profile estimations confirm that the changes in metabolic rate occur almost entirely during the stance phase (S3D and S4D Figs). Further analyzing the distribution of the costs over different muscle groups reveals that our estimations of the metabolic rate of the plantar flexors (57± 3% of the sum of all muscles with the musculoskeletal method and 41 ± 4% of the sum of all joints with the joint-space estimation method) were larger than the results from Umberger [20] (26 ± 10%, Table 4). Our estimations of the metabolic rate of the hip extensors (11 ± 1% with the musculoskeletal method and 24 ± 4% with the joint-space estimation) were lower than the results from Umberger (39 ± 6%). Other studies that used the Umberger estimation method appear to confirm the finding of a greater metabolic cost for the hip muscles than the ankle muscles [12,14], although the difference appears to be less pronounced in those works than in the study from Umberger [20]. These differences in the relative contributions of muscle groups likely contributed to the differences in the estimations of the time profile of metabolic rate during walking between studies.

**Table 4:** Estimated metabolic rate (% mean ± s.e.m.) of different muscle groups during level walking in our own analyses and other studies. For the musculoskeletal estimation method, we grouped the metabolic costs of different functions similar to [20] as follows: soleus, gastrocnemius medialis and gastrocnemius lateralis for the plantarflexors, vastus medialis for the knee extensors, gluteus maximus for the hip extensors, tibialis anterior for the dorsiflexors, and biceps femoris for the knee flexors. For the joint-space estimation method, we partitioned the metabolic costs for the ankle plantar flexors/dorsiflexors, knee extensors/flexors, and hip extensors/flexors based on when the joint moments were in the corresponding direction. The metabolic rate of muscle groups in the normal walking condition from the study by Jackson et al., [18] was obtained by applying the modified Umberger [20] estimation code from [68]. The data from Roberts et al., [23] were obtained from their supplementary data file for walking at 70% of the preferred walking speed. We selected this trial from their supplementary data because the walking speed is most similar to the walking speed from our study (1 m∙s^-1^. We obtained the unilateral metabolic rate from the right ankle knee and hip joints by entering the data from the right ankle knee and hip as inputs to their MATLAB code and entering signals with zeros for all non-lower limb joints and all joints on the left side of the body.

	Plantar flexors (%)	Knee extensors (%)	Hip extensors (%)	Dorsi flexors (%)	Knee flexors (%)	Hip flexors (%)
**Own musculoskeletal estimation**	57 ± 3.1	22 ± 2.5	11 ± 0.7	5 ± 1.4	5 ± 1.4	N/A
**Own joint-space estimation**	41 ± 3.5	7 ± 3.2	24 ± 4.3	1 ± 0.2	7 ± 1.3	20 ± 2.3
**Jackson et al.,** [18]	62 ± 4.0	23 ± 1.6	N/A	8 ± 1.0	7 ± 0.4	N/A
**Roberts et al.,** [23]	38 ± 36.9	9 ± 4.9	9 ± 7.8	4 ± 2.7	7 ± 3.5	33 ± 12.8
**Umberger** [20]	26 ± 10.1	18 ± 7.7	39 ± 16.1	17 ± 7.1		

A limitation of studies that estimate the metabolic rate time profile is that there is no direct measurement of the time profile to which the estimations can be compared, and the results depend on the chosen methods. Our data show that the estimations of the time profile can be different when using two different methods on the same data (i.e., an EMG-driven method in conjunction with muscle metabolic rate equations compared to a joint-space method). Overall, there are three main categories of methods to estimate the muscle states: forward simulations, inverse approaches, and EMG-driven approaches. Forward simulations predict the muscle behavior, joint kinematics, and kinetics that result from muscle excitations using a forward dynamics analysis of a multi-segment model with a foot-ground interaction model that estimates the ground reaction forces. These simulations can be designed to track human experimental data [79], or they can determine muscle excitations that optimize a specific objective (e.g., metabolic cost minimization in combination with a number of constraints, such as enforcing cyclic gait [20]). An advantage of this approach is that it can be used for predictive simulations of assistive devices that have not yet been tested. A disadvantage could be that the predicted activations, kinematics, and kinetics can sometimes be different from experimental data. Inverse approaches estimate the muscle state by distributing the moment from inverse dynamics over the muscles in a way that optimizes a physiologically realistic objective [12,16,58,80] such as minimizing the sum of squared activations [81]. Again, it is not known how universally valid this approach is, and other candidate objectives for computed muscle control have been proposed [82,83]. An advantage of this type of approach is that it can be used when specific EMG data are not available, for example, for specific deeper muscle layers that are hard to measure with surface EMG. Furthermore, EMG-driven approaches use recorded EMG in combination with prescribed kinematics to estimate the muscle states [18,56,57]. EMG-driven approaches offer the advantage of capturing the neural drive of the participants more realistically [37], but they require the most extensive experimental data (kinematics, EMG). Overall, it is likely that all three methods could result in different estimations. A case study was performed to evaluate to which extent using the same metabolic rate equations but with a different overall approach could affect metabolic time profile estimations. We compared a computed muscle control-based estimation to our EMG-driven estimation in one trial and found two different time profiles (S5 Fig). The metabolic rate equations can also have a substantial effect on the resulting metabolic rate estimations. A recent study from Koelewijn et al., [12] compared estimations with five different versions of metabolic equations on walking at downhill, level, and uphill grades with muscle-state estimations that were kept consistent across the different comparisons. Moreover, they included the equations from Umberger et al. [17], which are very similar to the equations used in our musculoskeletal method and the equations from Kim and Roberts, [22], which are similar to the joint-space method from [23]. They found a wide range of correlations of the different estimation methods compared to indirect calorimetry measurements, which confirms that muscle metabolic rate equations or joint-space-metabolic rate equations can have a considerable influence on estimated metabolic rates. Additionally, they found a slightly higher correlation and a regression slope closer to 1/1 for the Umberger et al., [17] equations compared to the joint-space equations. These findings are consistent with our results for the treadmill grade conditions that also show a higher correlation and regression slope closer to one with the muscle metabolic rate equations compared to the joint-space equations.

Although using EMG measurements for EMG-driven simulation ensured realistic activation, the fact that we did not model data from muscles that act predominantly outside the sagittal plane and the fact that we did not calibrate the muscle parameters to match the non-sagittal components of the hip moment could be limitations. Muscles that act in the frontal plane could contribute substantially to metabolic rate; for example, a study that models the effects of different exoskeleton configurations shows the potential benefits of assisting hip abduction [18]. To obtain a sense of how realistic our simulations were in addition to optimizing the agreement of the muscle-generated moments with the inverse dynamics moments, we plotted the muscle parameters, joint parameters, heat rates and metabolic rates for each muscle and joint in the condition with the level shoes on the level grade. Then, we compared these values versus those from similar metabolic estimation datasets [18,23] and literature from EMG, imaging, and tendon force measurement studies (S1 and S2 Figs). Our peak activations of the gastrocnemius (54%) were higher than those in the musculoskeletal simulation from Jackson et al. [18], but this appears to be closer to reference data of MVC-normalized gastrocnemius EMG (~80% [44]). The fiber length of the gastrocnemius shows a plateau during midstance before shortening at push-off, similar to data from ultrasound measurements [84]. The average time series of the fiber length of the soleus was slightly different from the Jackson et al., [18] simulation since it lengthens during the midstance phase. However, this behavior appears to be realistic based on a comparison with ultrasound data [71]. For more proximal muscles, there is less data on fiber kinematics, except the vastus medialis, for which the values from one experimental study show satisfactory agreement with our fiber length estimations [19,72,73]. The muscle activations for other muscles, joint angles and joint moments match well with the results from Jackson et al., [18] and Roberts et al., [23] studies.

Although there is no direct measurement of the time profile of metabolic rate during walking, we believe there could be opportunities to obtain additional information by conducting perturbation studies with devices such as exoskeletons [18,85,86] or tethers [87,88] that can be programmed to act during specific phases of the gait cycle. Optimizing coefficients in simulation models so that the estimations of the stride average metabolic rate match trends from “rich” datasets with perturbations to different parts of the gait cycle could potentially provide more confidence in estimations of the metabolic rate time profile. The ability to estimate the metabolic rate time profile with improved confidence levels could have a similar impact as transitions from average to dynamic measurements in other fields. For example, after dynamic plantar pressure measurement was developed [89], many clinical studies started to appear in the literature [90]. This technique is now used for various applications, such as foot-type classification [91], insole design [92], and physical therapy [93]. Estimating the metabolic rate time profile could have applications within the same categories (diagnosis [94], targeted exercise therapy [95], and assistive device optimization [96,97]).

This research demonstrated that new methods for estimating the metabolic rate time profile can match experimental estimations of the relative cost for the stance and swing phase, but the exact shape of the time profile is highly sensitive to the estimation method. Further improved estimations based on rich experimental datasets could lead to clinical applications and improve our understanding of the energetics of gait.

## Supporting information

S1 Fig Muscle-parameters, joint parameters, and metabolic rate estimations of the lower leg muscles and joints. Solid lines are the population average of our own data in the condition with level shoes on a level treadmill. Red dashed lines are the average results under normal walking conditions from the dataset from Jackson et al., [18]. The metabolic rate time profile in the normal walking condition was obtained from the study by Jackson et al., [18] by applying the modified Umberger [20] estimation code from [68]. Dashed blue lines are supplementary data from walking at 70% of the preferred walking speed from Roberts et al., [23]. We selected this trial from their supplementary data because the walking speed is most similar to the walking speed from our study (1 m∙s^-1^).
(TIFF)

S2 Fig Muscle parameters, joint parameters, and metabolic rate estimations of the upper leg muscles and joints. Full lines are the population average of our own data in the condition with level shoes on a level treadmill. Red dashed lines are the average results in the normal walking condition from the dataset from Jackson et al., [18]. The metabolic rate time profile in the normal walking condition was obtained from the study by Jackson et al. by applying the modified Umberger [20] estimation code from [68]. Dashed blue lines are supplementary data from walking at 70% of the preferred walking speed from Roberts et al., [23]. We selected this trial from their supplementary data because the walking speed is most similar to the walking speed from our study (1 m∙s^-1^).
(TIFF)

S3 Fig Effects of treadmill conditions on muscle parameters. **(a)** Hip metabolic rate. **(b)** Knee metabolic rate. **(c)** Ankle metabolic rate. **(d)** Total right side metabolic rate. **(e)** Total activation-maintenance heat rate. **(f)** Total shortening-lengthening heat rate. **(g)** Total fiber work. Lines represent subcomponents of the total metabolic rate estimated with the musculoskeletal method. Colors represent treadmill conditions. 100% on the vertical axis represents the stride average of the total metabolic rate of the right side.
(TIFF)

S4 Fig Effects of footwear conditions on muscle parameters. **(a)** Hip metabolic rate. **(b)** Knee metabolic rate. **(c)** Ankle metabolic rate. **(d)** Total right side metabolic rate. **(e)** Total activation-maintenance heat rate. **(f)** Total shortening-lengthening heat rate. **(g)** Total fiber work. Lines represent subcomponents of the total metabolic rate estimated with the musculoskeletal method. Colors represent treadmill conditions. 100% on the vertical axis represents the stride average of the total metabolic rate of the right side.
(TIFF)

S5 Fig Supplementary computed muscle control comparison. Comparison of estimation of the time profile of metabolic rate using EMG-driven musculoskeletal method versus computed muscle control (CMC) data. The EMG-driven timeseries is a metabolic time profile of one participant during level walking, obtained with the model, musculoskeletal method, and muscle metabolic rate equations that are used in the musculoskeletal method in the main text. The computed muscle control time series is a metabolic time profile of the same participant and walking condition, obtained with the same muscle metabolic rate equations but with a different model and general approach to obtain the states of the muscles. For the computed muscle control driven timeseries, we used the computed muscle control tool in combination with the default model (Gait2392) in OpenSim to obtain the muscle states from 46 lower leg muscles per side. This comparison shows that metabolic time profile estimations can be different even when the same muscle metabolic rate equations are used but with different models and general approaches to obtain muscle state data.
(TIFF)

S1 Table Raw metabolic rate data in absolute units (W kg^-1^). Treadmill grade conditions, shoe inclination conditions and normal walking condition. Rows indicate participants. Columns include different estimation methods and indirect calorimetry.
(PDF)

S2 Table Changes in metabolic rate in percent of normal walking condition. Treadmill grade conditions and shoe inclination conditions. Columns include different estimation methods and indirect calorimetry. Rows represent mean and inter-participant s.e.m.
(PDF)

S1 File MATLAB data file named “Data_MetabolicTimeProfileEstimation.mat” with temporal, kinematic, kinetic, muscle, and metabolic data for both metabolic cost estimation conditions for all participants and conditions. A text file named “Data_MetabolicTimeProfileEstimation—readme.txt” with an explanation on data organization is included.
(ZIP)

## References

[1] Bertram JEA, Ruina A. Multiple walking speed-frequency relations are predicted by constrained optimization. J Theor Biol. 2001;209:445–453. 10.1006/jtbi.2001.2279 11319893

[2] Zarrugh MY, Todd FN, Ralston HJ. Optimization of energy expenditure during level walking. Eur J Appl Physiol Occup Physiol. 1974;33:293–306. 10.1007/BF00430237 4442409

[3] Molen NH, Rozendal RH, Boon W. Graphic representation of the relationship between oxygen-consumption and characteristics of normal gait of the human male. Proc K Ned Akad Wet C. 1972;75:305–14. Available: http://europepmc.org/abstract/MED/4263757 4263757

[4] Ralston HJ. Energy-speed relation and optimal speed during level walking. Int Z Angew Physiol. 1958;17:277–83. Available: http://www.ncbi.nlm.nih.gov/pubmed/13610523 10.1007/BF00698754 13610523

[5] Selinger JC, O’Connor SM, Wong JD, Donelan JM. Humans Can Continuously Optimize Energetic Cost during Walking. Curr Biol. Elsevier Ltd; 2015;25:2452–2456. 10.1016/j.cub.2015.08.016 26365256

[6] Waters RL, Perry J, Antonelli D, Hislop H. Energy cost of walking of amputees: the influence of level of amputation. J Bone Joint Surg Am. 1976;58:42–6. Available: http://www.ncbi.nlm.nih.gov/pubmed/1249111 1249111

[7] Platts MM, Rafferty D, Paul L. Metabolic cost of overground gait in younger stroke patients and healthy controls. Med Sci Sports Exerc. 2006;38:1041–1046. 10.1249/01.mss.0000222829.34111.9c 16775542

[8] Rose J, Gamble JG, Burgos A, Medeiros J, Haskell WL. Energy expenditure index of walking for normal children and for children with cerebral palsy. Dev Med Child Neurol. 1990;32:333–40. 10.1111/j.1469-8749.1990.tb16945.x 2332124

[9] Baker JS, McCormick MC, Robergs RA. Interaction among Skeletal Muscle Metabolic Energy Systems during Intense Exercise. J Nutr Metab. 2010;2010:1–13. 10.1155/2010/905612 21188163 PMC3005844

[10] Bhargava LJ, Pandy MG, Anderson FC. A phenomenological model for estimating metabolic energy consumption in muscle contraction. J Biomech. 2004;37:81–88. 10.1016/s0021-9290(03)00239-2 14672571

[11] Houdijk H, Bobbert MF, De Haan A. Evaluation of a Hill based muscle model for the energy cost and efficiency of muscular contraction. J Biomech. 2006;39:536–543. 10.1016/j.jbiomech.2004.11.033 16389094

[12] Koelewijn AD, Heinrich D, van den Bogert AJ. Metabolic cost calculations of gait using musculoskeletal energy models, a comparison study. Grabowski A, editor. PLoS One. 2019;14:e0222037 10.1371/journal.pone.0222037 31532796 PMC6750598

[13] Lichtwark GA, Wilson AM. A modified Hill muscle model that predicts muscle power output and efficiency during sinusoidal length changes. J Exp Biol. 2005;208:2831–2843. 10.1242/jeb.01709 16043588

[14] Miller RH. A comparison of muscle energy models for simulating human walking in three dimensions. J Biomech. Elsevier; 2014;47:1373–1381. 10.1016/j.jbiomech.2014.01.049 24581797

[15] Minetti AE, Alexander RMN. A theory of metabolic costs for bipedal gaits. J Theor Biol. 1997;186:467–476. 10.1006/jtbi.1997.0407 9278722

[16] Uchida TK, Hicks JL, Dembia CL, Delp SL. Stretching your energetic budget: How tendon compliance affects the metabolic cost of running. PLoS One. 2016;11:1–19. 10.1371/journal.pone.0150378 26930416 PMC4773147

[17] Umberger BR, Gerritsen KGM, Martin PE. A model of human muscle energy expenditure. Comput Methods Biomech Biomed Engin. 2003;6:99–111. 10.1080/1025584031000091678 12745424

[18] Jackson RW, Dembia CL, Delp SL, Collins SH. Muscle-tendon mechanics explain unexpected effects of exoskeleton assistance on metabolic rate during walking. J Exp Biol. 2017;220:2082–2095. 10.1242/jeb.150011 28341663 PMC6514464

[19] Markowitz J, Herr H. Human Leg Model Predicts Muscle Forces, States, and Energetics during Walking. PLoS Comput Biol. 2016;12 10.1371/journal.pcbi.1004912 27175486 PMC4866735

[20] Umberger BR. Stance and swing phase costs in human walking. J R Soc Interface. 2010;7:1329–40. 10.1098/rsif.2010.0084 20356877 PMC2894890

[21] Hill A V. The heat of shortening and the dynamic constants of muscle. Proc R Soc London Ser B—Biol Sci. 1938;126:136–195. 10.1098/rspb.1938.0050

[22] Kim JH, Roberts D. A joint-space numerical model of metabolic energy expenditure for human multibody dynamic system. Int j numer method biomed eng. 2015;31:e02721 10.1002/cnm.2721 25914404

[23] Roberts D, Hillstrom H, Kim JH. Instantaneous metabolic cost of walking: Joint-space dynamic model with subject-specific heat rate. PLoS One. 2016;11:14–16. 10.1371/journal.pone.0168070 28030598 PMC5193358

[24] Arellano CJ, Kram R. Partitioning the metabolic cost of human running: A task-by-task approach. Integr Comp Biol. 2014;54:1084–1098. 10.1093/icb/icu033 24838747 PMC4296200

[25] Faraji S, Wu AR, Ijspeert AJ. A simple model of mechanical effects to estimate metabolic cost of human walking. Sci Rep. Springer US; 2018;8:1–12. 10.1038/s41598-018-29429-z 30030539 PMC6054663

[26] Grabowski A, Farley CT, Kram R. Independent metabolic costs of supporting body weight and accelerating body mass during walking. J Appl Physiol. 2005;98:579–83. 10.1152/japplphysiol.00734.2004 15649878

[27] Griffin TM, Roberts TJ, Kram R. Metabolic cost of generating muscular force in human walking: Insights from load-carrying and speed experiments. J Appl Physiol. 2003;95:172–183. 10.1152/japplphysiol.00944.2002 12794096

[28] Doke J, Donelan JM, Kuo AD. Mechanics and energetics of swinging the human leg. J Exp Biol. 2005;208:439–45. 10.1242/jeb.01408 15671332

[29] Gottschall JS, Kram R. Energy cost and muscular activity required for leg swing during walking. J Appl Physiol. 2005;99:23–30. 10.1152/japplphysiol.01190.2004 16036902

[30] Koelewijn AD, Dorschky E, van den Bogert AJ. A metabolic energy expenditure model with a continuous first derivative and its application to predictive simulations of gait. Comput Methods Biomech Biomed Engin. Taylor & Francis; 2018;21:521–531. 10.1080/10255842.2018.1490954 30027769

[31] Stagni R, Fantozzi S, Cappello A, Leardini A. Quantification of soft tissue artefact in motion analysis by combining 3D fluoroscopy and stereophotogrammetry: a study on two subjects. Clin Biomech. 2005;20:320–329. 10.1016/j.clinbiomech.2004.11.012 15698706

[32] Tsushima H, Morris ME, McGinley J. Test-Retest Reliability and Inter-Tester Reliability of Kinematic Data from a Three-Dimensional Gait Analysis System. J Japanese Phys Ther Assoc. 2003;6:9–17. 10.1298/jjpta.6.9 25792928 PMC4316510

[33] Collins SH, Bruce Wiggin M, Sawicki GS. Reducing the energy cost of human walking using an unpowered exoskeleton. Nature. 2015;522:212–215. 10.1038/nature14288 25830889 PMC4481882

[34] Mochon S, McMahon TA. Ballistic walking. J Biomech. 1980;13:49–57. 10.1016/0021-9290(80)90007-x 7354094

[35] Kuo AD. Energetics of actively powered locomotion using the simplest walking model. J Biomech Eng. 2002;124:113–120. 10.1115/1.1427703 11871597

[36] Antonellis P, Frederick CM, Gonabadi AM, Malcolm P. Modular footwear that partially offsets downhill or uphill grades minimizes the metabolic cost of human walking. R Soc open Sci. 2020;7:191527 10.1098/rsos.191527 32257319 PMC7062060

[37] Buchanan TS, Lloyd DG, Manal K, Besier TF. Neuromusculoskeletal modeling: Estimation of muscle forces and joint moments and movements from measurements of neural command. J Appl Biomech. 2004;20:367–395. 10.1123/jab.20.4.367 16467928 PMC1357215

[38] Hicks JL, Uchida TK, Seth A, Rajagopal A, Delp SL. Is My Model Good Enough? Best Practices for Verification and Validation of Musculoskeletal Models and Simulations of Movement. J Biomech Eng. 2015;137 10.1115/1.4029304 25474098 PMC4321112

[39] Dorn TW, Wang JM, Hicks JL, Delp SL. Predictive simulation generates human adaptations during loaded and inclined walking. PLoS One. 2015;10:1–16. 10.1371/journal.pone.0121407 25830913 PMC4382289

[40] Franz JR, Kram R. The effects of grade and speed on leg muscle activations during walking. Gait Posture. 2012;35:143–147. 10.1016/j.gaitpost.2011.08.025 21962846 PMC3262943

[41] Lay AN, Hass CJ, Gregor RJ. The effects of sloped surfaces on locomotion: A kinematic and kinetic analysis. J Biomech. 2006;39:1621–1628. 10.1016/j.jbiomech.2005.05.005 15990102

[42] Kadaba MP, Ramakrishnan HK, Wootten ME. Measurement of lower extremity kinematics during level walking. J Orthop Res. 1990;8:383–392. 10.1002/jor.1100080310 2324857

[43] Brockway JM. Derivation of formulae used to calculate energy expenditure in man. Hum Nutr Clin Nutr. 1987;41:463–71. Available: http://www.ncbi.nlm.nih.gov/pubmed/3429265 3429265

[44] Perry J, Burnfield JM. Gait analysis: normal and pathological function. Journal of Sports Science and Medicine. 2010 10.1302/0301-620X.92B8.0921184a

[45] Winter DA. The Biomechanics and Motor Control of Human Gait: Normal, Elderly and Pathological. University of Waterloo Press; 1991.

[46] Seth A, Hicks JL, Uchida TK, Habib A, Dembia CL, Dunne JJ, et al OpenSim: Simulating musculoskeletal dynamics and neuromuscular control to study human and animal movement. PLoS Comput Biol. 2018;14 10.1371/journal.pcbi.1006223 30048444 PMC6061994

[47] Delp SL, Anderson FC, Arnold AS, Loan P, Habib A, John CT, et al OpenSim: Open-source software to create and analyze dynamic simulations of movement. IEEE Trans Biomed Eng. 2007;54:1940–1950. 10.1109/TBME.2007.901024 18018689

[48] Rajagopal A, Dembia CL, Demers MS, Delp DD, Hicks JL, Delp SL. Full body musculoskeletal model for muscle- driven simulation of human gait. 2015; 10.1109/TBME.2016.2586891 PMC5507211 27392337

[49] Yong JR, Dembia CL, Silder A, Jackson RW, Fredericson M, Delp SL. Foot strike pattern during running alters muscle-tendon dynamics of the gastrocnemius and the soleus. bioRxiv Prepr. 2019; 1–25. 10.1038/s41598-020-62464-3 PMC7125118 32245985

[50] Millard M, Uchida T, Seth A, Delp SL. Flexing computational muscle: modeling and simulation of musculotendon dynamics. J Biomech Eng. 2013;135:021005 10.1115/1.4023390 23445050 PMC3705831

[51] Arnold EM, Hamner SR, Seth A, Millard M, Delp SL. How muscle fiber lengths and velocities affect muscle force generation as humans walk and run at different speeds. J Exp Biol. 2013;216:2150–2160. 10.1242/jeb.075697 23470656 PMC3656509

[52] Handsfield GG, Meyer CH, Hart JM, Abel MF, Blemker SS. Relationships of 35 lower limb muscles to height and body mass quantified using MRI. J Biomech. 2014;47:631–638. 10.1016/j.jbiomech.2013.12.002 24368144

[53] Ward SR, Tomiya A, Regev GJ, Thacker BE, Benzl RC, Kim CW, et al Passive mechanical properties of the lumbar multifidus muscle support its role as a stabilizer. J Biomech. 2009;42:1384–1389. 10.1016/j.jbiomech.2008.09.042 19457491 PMC2752430

[54] Farris DJ. PrescribeMotionInModel.m. 2017. Available: https://github.com/opensim-org/opensim-core/blob/master/Bindings/Java/Matlab/examples/prescribeMotionInModel.m

[55] MetabolicsSlowTwitchRatios_Gait2392. 2017. Available: https://github.com/opensim-org/opensim-models/blob/master/Tutorials/Design_to_Reduce_Metabolic_Cost/Scripts/metabolicsSlowTwitchRatios_Gait2392.txt

[56] Falisse A, Van Rossom S, Jonkers I, De Groote F. EMG-Driven Optimal Estimation of Subject-SPECIFIC Hill Model Muscle-Tendon Parameters of the Knee Joint Actuators. IEEE Trans Biomed Eng. 2017;64:2253–2262. 10.1109/TBME.2016.2630009 27875132

[57] Lloyd DG, Besier TF. An EMG-driven musculoskeletal model to estimate muscle forces and knee joint moments in vivo. J Biomech. 2003;36:765–776. 10.1016/s0021-9290(03)00010-1 12742444

[58] De Groote F, Kinney AL, Rao A V., Fregly BJ. Evaluation of Direct Collocation Optimal Control Problem Formulations for Solving the Muscle Redundancy Problem. Ann Biomed Eng. 2016;44:2922–2936. 10.1007/s10439-016-1591-9 27001399 PMC5043004

[59] Scovil CY, Ronsky JL. Sensitivity of a Hill-based muscle model to perturbations in model parameters. J Biomech. 2006;39:2055–2063. 10.1016/j.jbiomech.2005.06.005 16084520

[60] Redl C, Gfoehler M, Pandy MG. Sensitivity of muscle force estimates to variations in muscle-tendon properties. Hum Mov Sci. 2007;26:306–319. 10.1016/j.humov.2007.01.008 17343945

[61] De Groote F, Van Campen A, Jonkers I, De Schutter J. Sensitivity of dynamic simulations of gait and dynamometer experiments to hill muscle model parameters of knee flexors and extensors. J Biomech. 2010;43:1876–1883. 10.1016/j.jbiomech.2010.03.022 20392450

[62] Ackland DC, Lin YC, Pandy MG. Sensitivity of model predictions of muscle function to changes in moment arms and muscle-tendon properties: A Monte-Carlo analysis. J Biomech. 2012;45:1463–1471. 10.1016/j.jbiomech.2012.02.023 22507351

[63] Silder A, Whittington B, Heiderscheit B, Thelen DG. Identification of passive elastic joint moment-angle relationships in the lower extremity. J Biomech. 2007;40:2628–2635. 10.1016/j.jbiomech.2006.12.017 17359981 PMC2020832

[64] Nouri Damghani M, Mohammadzadeh Gonabadi A. Multi-Objective Optimization of Kinematic Characteristics of Geneva Mechanism Using High-Tech Optimization Methods. Mech Mater Sci Eng. 2017;8 10.2412/mmse.26.65.331

[65] Mohammadzadeh A, Ghoddoosian A, Nouri Damghani M. Balancing of the Flexible Rotors with Particle Swarm Optimization Method. Int Rev Mech Eng. 2011;5:490–496.

[66] Neptune RR, Sasaki K. Ankle plantar flexor force production is an important determinant of the preferred walk-to-run transition speed. J Exp Biol. 2005;208:799–808. 10.1242/jeb.01435 15755878

[67] Pandy MG, Andriacchi TP. Muscle and Joint Function in Human Locomotion. Annu Rev Biomed Eng. 2010;12:401–433. 10.1146/annurev-bioeng-070909-105259 20617942

[68] Dorn T, Uchida TK. OpenSim: UchidaUmberger2010MuscleMetabolicsProbe.cpp. 2014. Available: https://github.com/opensim-org/opensim-metabolicsprobes/blob/master/UchidaUmberger2010MuscleMetabolicsProbe.cpp

[69] Mendez J, Keys A. Density and Composition of Mammalian Muscle. Metab Exp. 1960;9:184–188. Available: isi:A1960WP37300007

[70] González-Alonso J, Quistorff B, Krustrup P, Bangsbo J, Saltin B. Heat production in human skeletal muscle at the onset of intense dynamic exercise. J Physiol. 2000;524 Pt 2:603–15. 10.1111/j.1469-7793.2000.00603.x 10766936 PMC2269891

[71] Ishikawa M, Komi P V, Grey MJ, Lepola V, Bruggemann G-P. Muscle-tendon interaction and elastic energy usage in human walking. J Appl Physiol. 2005;99:603–8. 10.1152/japplphysiol.00189.2005 15845776

[72] Chleboun GS, France AR, Crill MT, Braddock HK, Howell JN. In vivo measurement of fascicle length and pennation angle of the human biceps femoris muscle. Cells Tissues Organs. 2001;169:401–9. 10.1159/000047908 11490120

[73] Chleboun GS, Busic AB, Graham KK, Stuckey HA. Fascicle length change of the human tibialis anterior and vastus lateralis during walking. J Orthop Sports Phys Ther. 2007;37:372–9. 10.2519/jospt.2007.2440 17710906

[74] Lichtwark GA. In vivo mechanical properties of the human Achilles tendon during one-legged hopping. J Exp Biol. 2005;208:4715–4725. 10.1242/jeb.01950 16326953

[75] Tukey John W. Exploratory Data Analysis. Pearson; 1977.

[76] Bakdash JZ, Marusich LR. Repeated measures correlation. Front Psychol. 2017;8 10.3389/fpsyg.2017.00456 28439244 PMC5383908

[77] Silder A, Heiderscheit B, Thelen DG. Active and passive contributions to joint kinetics during walking in older adults. J Biomech. 2008;41:1520–1527. 10.1016/j.jbiomech.2008.02.016 18420214 PMC2702713

[78] Lewis CL, Ferris DP. Invariant hip moment pattern while walking with a robotic hip exoskeleton. J Biomech. 2011;44:789–793. 10.1016/j.jbiomech.2011.01.030 21333995 PMC3075111

[79] Thelen DG, Anderson FC. Using computed muscle control to generate forward dynamic simulations of human walking from experimental data. J Biomech. 2006;39:1107–1115. 10.1016/j.jbiomech.2005.02.010 16023125

[80] Thelen DG. Adjustment of muscle mechanics model parameters to simulate dynamic contractions in older adults. J Biomech Eng. 2003;125:70–77. 10.1115/1.1531112 12661198

[81] Crowninshield RD, Brand RA. A physiologically based criterion of muscle force prediction in locomotion. J Biomech. 1981;14:793–801. 10.1016/0021-9290(81)90035-x 7334039

[82] Wang JM, Hamner SR, Delp SL, Koltun V. Optimizing Locomotion Controllers Using Biologically-Based Actuators and Objectives. ACM Trans Graph. 2012;31 10.1145/2185520.2185521 26251560 PMC4523558

[83] Ackermann M, van den Bogert AJ. Optimality principles for model-based prediction of human gait. J Biomech. 2010;43:1055–60. 10.1016/j.jbiomech.2009.12.012 20074736 PMC2849893

[84] Lichtwark GA, Bougoulias K, Wilson AM. Muscle fascicle and series elastic element length changes along the length of the human gastrocnemius during walking and running. J Biomech. 2007;40:157–64. 10.1016/j.jbiomech.2005.10.035 16364330

[85] Farris DJ, Hicks JL, Delp SL, Sawicki GS. Musculoskeletal modelling deconstructs the paradoxical effects of elastic ankle exoskeletons on plantar-flexor mechanics and energetics during hopping. J Exp Biol. 2014;217:4018–4028. 10.1242/jeb.107656 25278469 PMC4229366

[86] Antonellis P, Galle S, De Clercq D, Malcolm P. Altering gait variability with an ankle exoskeleton. PLoS One. 2018;13:e0205088 10.1371/journal.pone.0205088 30356309 PMC6200209

[87] Gonabadi AM, Antonellis P, Malcolm P. A System for Simple Robotic Walking Assistance With Linear Impulses at the Center of Mass. IEEE Trans Neural Syst Rehabil Eng. 2020;28:1353–1362. 10.1109/TNSRE.2020.2988619 32340953 PMC7404782

[88] Simha SN, Wong JD, Selinger JC, Donelan JM. A Mechatronic System for Studying Energy Optimization During Walking. IEEE Trans Neural Syst Rehabil Eng. 2019;27:1416–1425. 10.1109/TNSRE.2019.2917424 31107655

[89] Cavanagh PR, Ae M. A technique for the display of pressure distributions beneath the foot. J Biomech. 1980;13:69–75. 10.1016/0021-9290(80)90180-3 7364781

[90] Hughes J. The clinical use of pedobarography. Acta Orthopaedica Belgica. 1993 pp. 10–16. 8484313

[91] De Cock A, Willems T, Witvrouw E, Vanrenterghem J, De Clercq D. A functional foot type classification with cluster analysis based on plantar pressure distribution during jogging. Gait Posture. 2006;23:339–347. 10.1016/j.gaitpost.2005.04.011 15990311

[92] Owings TM, Woerner JL, Frampton JD, Cavanagh PR, Botek G. Custom therapeutic insoles based on both foot shape and plantar pressure measurement provide enhanced pressure relief. Diabetes Care. 2008;31:839–844. 10.2337/dc07-2288 18252899

[93] Huffer D, Hing W, Newton R, Clair M. Strength training for plantar fasciitis and the intrinsic foot musculature: A systematic review. Phys Ther Sport. 2017;24:44–52. 10.1016/j.ptsp.2016.08.008 27692740

[94] Rao KS. Understanding elevated metabolic cost of asymmetric walking. Johns Hopkins University. 2019 Available: http://jhir.library.jhu.edu/handle/1774.2/61681

[95] Reisman D, Binder-Macleod S, Farquhar W. Changes in metabolic cost of transport following locomotor training poststroke. Top Stroke Rehabil. 2013;20:161–170. 10.1310/tsr2002-161 23611857 PMC4104066

[96] Awad LN, Bae J, O’Donnell K, De Rossi SMM, Hendron K, Sloot LH, et al A soft robotic exosuit improves walking in patients after stroke. Sci Transl Med. 2017;9 10.1126/scitranslmed.aai9084 28747517

[97] Lerner ZF, Gasparri GM, Bair MO, Lawson JL, Luque J, Harvey TA, et al An untethered ankle exoskeleton improves walking economy in a pilot study of individuals with cerebral palsy. IEEE Trans Neural Syst Rehabil Eng. IEEE; 2018;26:1985–1993. 10.1109/TNSRE.2018.2870756 30235140 PMC6217810

