# Supplementary figures and images for: Differences between joint-space and musculoskeletal estimations of metabolic rate time profiles
Source: PLoS Comput Biol. 2020 Oct 28;16(10):e1008280. doi: 10.1371/journal.pcbi.1008280 (PMC7592801; doi:10.1371/journal.pcbi.1008280)

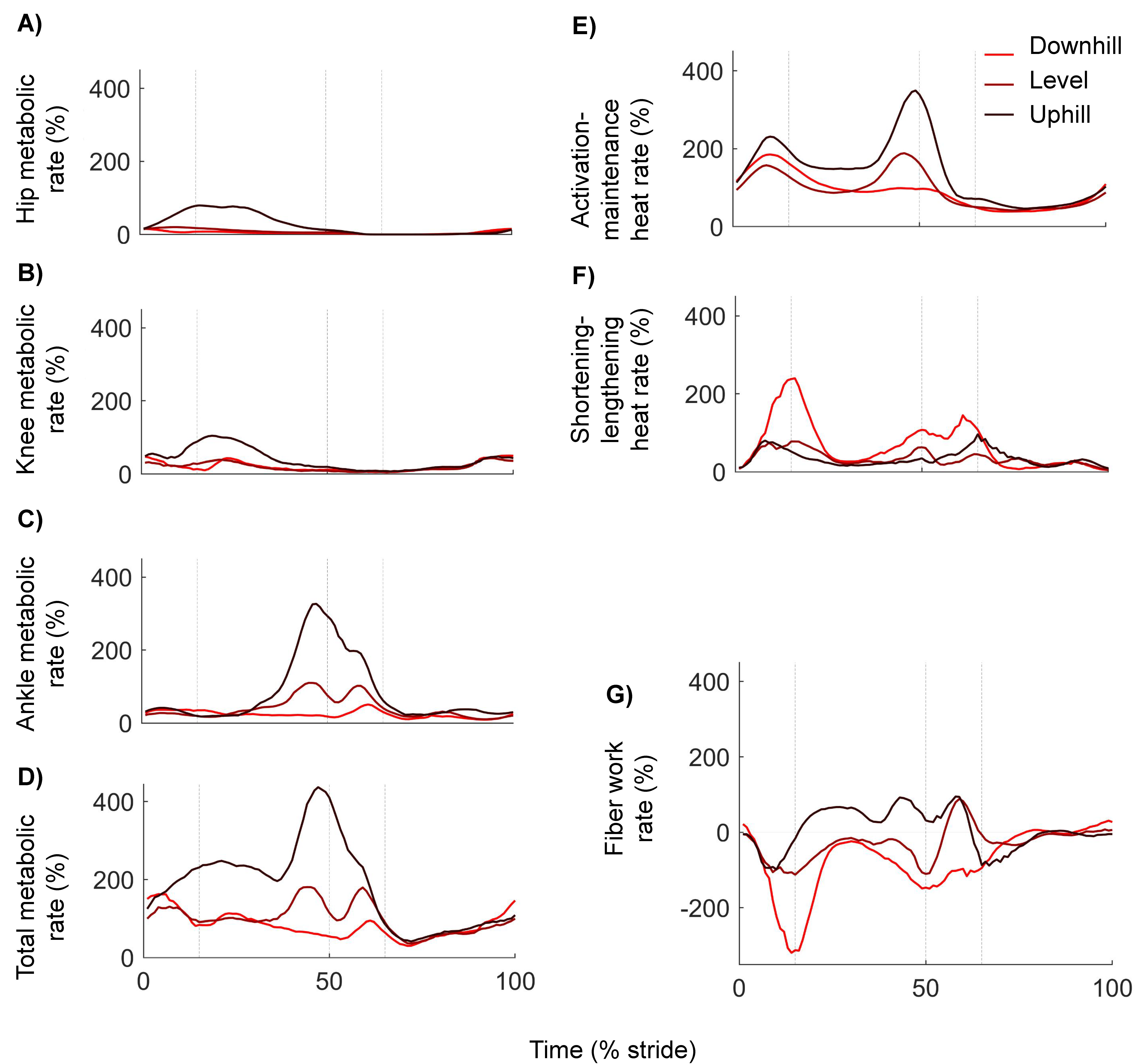

Supplement: S3 Fig — (a) Hip metabolic rate. (b) Knee metabolic rate. (c) Ankle metabolic rate. (d) Total right side metabolic rate. (e) Total activation-maintenance heat rate. (f) Total shortening-lengthening heat rate. (g) Total fiber work. Lines represent subcomponents of the total metabolic rate estimated with the musculoskeletal method. Colors represent treadmill conditions. 100% on the vertical axis represents the stride average of the total metabolic rate of the right side. (TIFF) [file pcbi.1008280.s003.tiff]

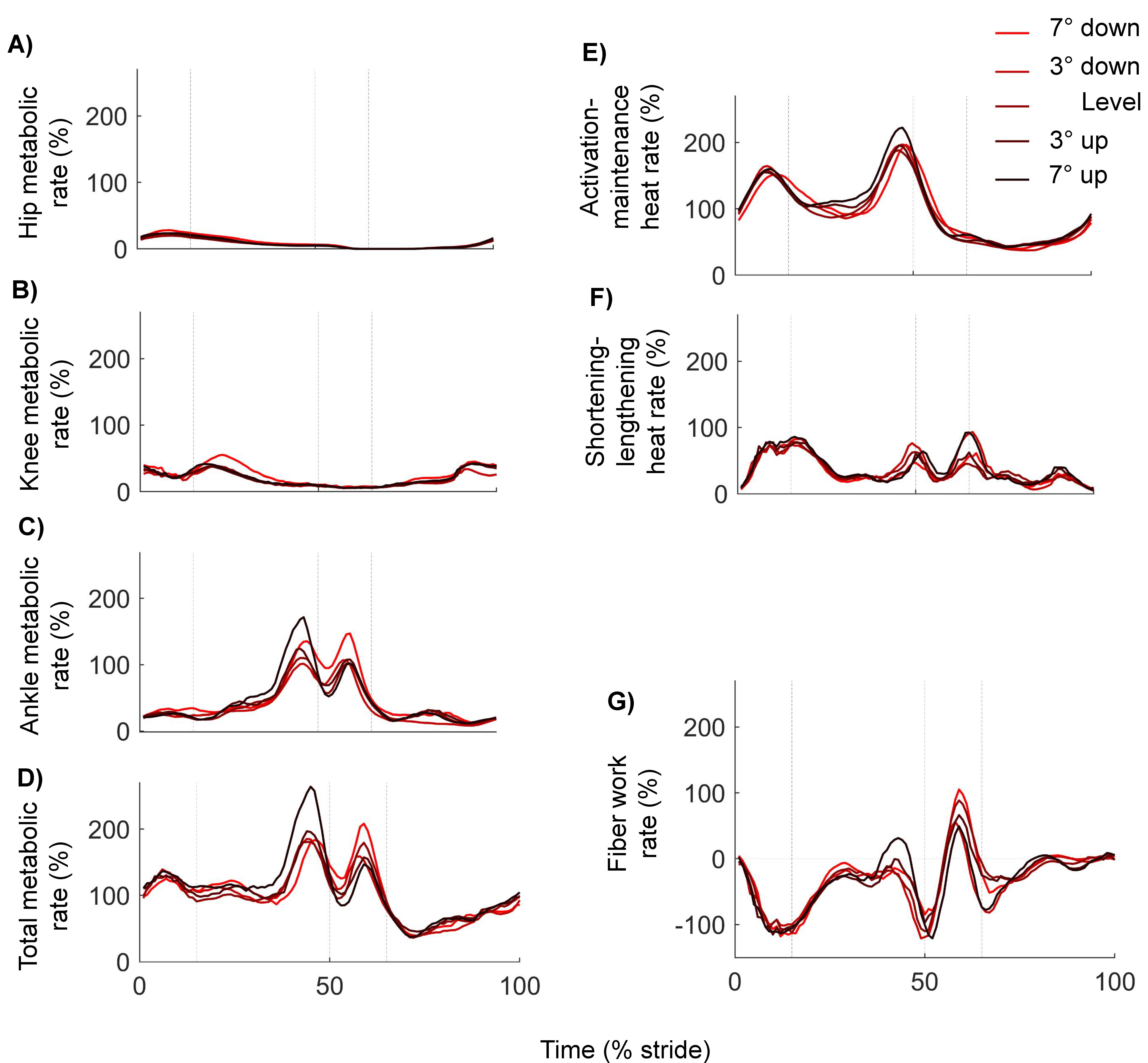

Supplement: S4 Fig — (a) Hip metabolic rate. (b) Knee metabolic rate. (c) Ankle metabolic rate. (d) Total right side metabolic rate. (e) Total activation-maintenance heat rate. (f) Total shortening-lengthening heat rate. (g) Total fiber work. Lines represent subcomponents of the total metabolic rate estimated with the musculoskeletal method. Colors represent treadmill conditions. 100% on the vertical axis represents the stride average of the total metabolic rate of the right side. (TIFF) [file pcbi.1008280.s004.tiff]

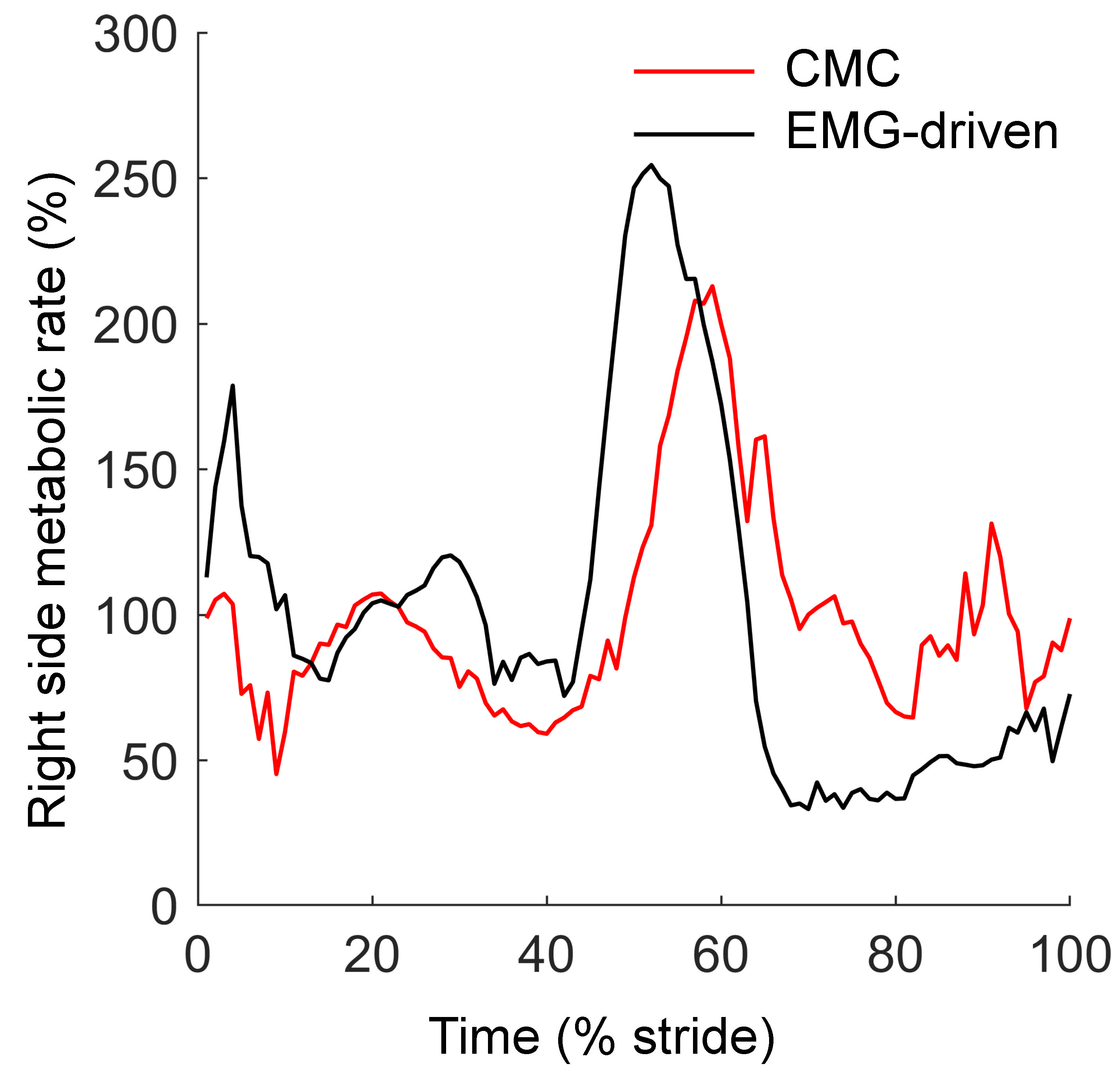

Supplement: S5 Fig — Comparison of estimation of the time profile of metabolic rate using EMG-driven musculoskeletal method versus computed muscle control (CMC) data. The EMG-driven timeseries is a metabolic time profile of one participant during level walking, obtained with the model, musculoskeletal method, and muscle metabolic rate equations that are used in the musculoskeletal method in the main text. The computed muscle control time series is a metabolic time profile of the same participant and walking condition, obtained with the same muscle metabolic rate equations but with a different model and general approach to obtain the states of the muscles. For the computed muscle control driven timeseries, we used the computed muscle control tool in combination with the default model (Gait2392) in OpenSim to obtain the muscle states from 46 lower leg muscles per side. This comparison shows that metabolic time profile estimations can be different even when the same muscle metabolic rate equations are used but with different models and general approaches to obtain muscle state data. (TIFF) [file pcbi.1008280.s005.tiff]
