# Supplementary material for: Differences between joint-space and musculoskeletal estimations of metabolic rate time profiles
Source: PLoS Comput Biol. 2020 Oct 28;16(10):e1008280. doi: 10.1371/journal.pcbi.1008280 (PMC7592801; doi:10.1371/journal.pcbi.1008280)
Supplement: S1 Table — Treadmill grade conditions, shoe inclination conditions and normal walking condition. Rows indicate participants. Columns include different estimation methods and indirect calorimetry. (PDF) [file pcbi.1008280.s006.pdf]

S1 Table. Raw metabolic rate data in absolute units (W kg^{-1}). Treadmill grade conditions, shoe inclination conditions and normal walking condition. Rows indicate participants. Columns include different estimation methods and indirect calorimetry.

	Treadmill grade conditions						Shoe inclination conditions												Level treadmill grade and level shoe condition		
	-6° Downhill			+6° Uphill			-7° Downward			-3° Downward			+3° Upward			+7° Upward					
Participant	Musculo-skeletal method	Joint-space method	Indirect calori-metry	Musculo-skeletal method	Joint-space method	Indirect calorimetry	Musculo-skeletal method	Joint-space method	Indirect calori-metry	Musculo-skeletal method	Joint-space method	Indirect calori-metry	Musculo-skeletal method	Joint-space method	Indirect calori-metry	Musculo-skeletal method	Joint-space method	Indirect calori-metry	Musculo-skeletal method	Joint-space method	Indirect calori-metry
1	1.01	6.06	1.90	2.03	2.99	6.28	1.17	2.44	3.03	1.09	2.63	2.84	1.11	2.66	3.22	1.12	2.72	2.95	1.03	2.53	3.10
2	0.88	1.97	1.82	1.84	4.03	7.33	1.05	2.48	3.61	1.00	2.33	3.40	1.08	2.43	3.62	1.12	2.60	3.63	1.00	2.70	3.07
3	0.98	1.64	2.11	2.05	4.29	6.55	1.22	2.41	4.16	N/A	N/A	3.17	1.08	2.49	3.17	1.18	2.41	3.38	1.15	2.26	3.04
4	0.62	1.95	2.15	1.48	3.52	6.55	0.78	2.41	3.13	0.82	2.57	3.09	0.89	2.52	3.19	1.05	2.68	2.76	0.82	2.58	3.14
5	0.76	2.13	1.76	1.61	4.14	6.49	1.37	2.68	2.85	0.95	2.60	2.78	1.01	3.16	2.81	1.02	2.89	2.90	0.91	2.83	2.74
6	0.78	1.46	2.87	1.42	3.42	7.64	0.95	2.30	5.36	0.81	2.23	3.54	0.82	2.49	3.01	0.91	2.38	5.45	0.94	2.18	4.73
