# Supplementary material for: Differences between joint-space and musculoskeletal estimations of metabolic rate time profiles
Source: PLoS Comput Biol. 2020 Oct 28;16(10):e1008280. doi: 10.1371/journal.pcbi.1008280 (PMC7592801; doi:10.1371/journal.pcbi.1008280)
Supplement: S2 Table — Treadmill grade conditions and shoe inclination conditions. Columns include different estimation methods and indirect calorimetry. Rows represent mean and inter-participant s.e.m. (PDF) [file pcbi.1008280.s007.pdf]

S2 Table. Changes in metabolic rate in percent of normal walking condition. Treadmill grade conditions and shoe inclination conditions. Columns include different estimation methods and indirect calorimetry. Rows represent mean and inter-participant s.e.m.

	Treadmill grade conditions						Shoe inclination conditions											
	-6° Downhill			+6° Uphill			-7° Downward			-3° Downward			+3° Upward			+7° Upward		
	Musculo-skeletal method	Joint-space method	Indirect calori-metry	Musculo-skeletal method	Joint-space method	Indirect calorimetry	Musculo-skeletal method	Joint-space method	Indirect calori-metry	Musculo-skeletal method	Joint-space method	Indirect calori-metry	Musculo-skeletal method	Joint-space method	Indirect calori-metry	Musculo-skeletal method	Joint-space method	Indirect calori-metry
Mean	-17	-27	-36	83	49	111	4.2	-1.9	11.6	-0.4	-3.2	-3.1	3.0	4.8	3.0	10.1	4.3	5.6
s.e.m.	2.1	1.6	1.7	3.6	9.7	11.5	2.9	2.6	6.0	3.4	3.3	5.1	3.9	3.8	0.6	4.3	1.9	4.8
